# Hyperoxia but not high tidal volume contributes to ventilator-induced lung injury in healthy mice

**DOI:** 10.1186/s12890-023-02626-x

**Published:** 2023-09-20

**Authors:** Yong Xu, Yu Li, Da Zhai, Chen Yan, Jingyan Liang, Taiga Ichinomiya, Tetsuya Hara, Chiaki Inadomi, Tao-Sheng Li

**Affiliations:** 1 Department of Stem Cell Biology, Atomic Bomb Disease Institute, Nagasaki University, 1-12-4 Sakamoto, Nagasaki, 852-8523 Japan; 2 Department of Stem Cell Biology, Nagasaki University Graduate School of Biomedical Sciences, 1-12-4 Sakamoto, Nagasaki, 852-8523 Japan; 3 Department of Anesthesiology, The Second Affiliated Hospital of Nanchang University, Nanchang City, 330006 Jiangxi Province China; 4 Institute of Translational Medicine, Medical College, Yangzhou University, Yangzhou, 225000 Jiangsu P.R. China; 5 Department of Anesthesiology and Intensive Care Medicine, Nagasaki University Graduate School of Biomedical Sciences, 1-7-1 Sakamoto, Nagasaki, 852-8501 Japan

**Keywords:** Mechanical ventilation, Tidal volume, Oxidative damage, Inflammatory response, Mechanotransduction

## Abstract

**Background:**

Mechanical ventilation is a supportive therapy used to maintain respiratory function in several clinical and surgical cases but is always accompanied by lung injury risk due to improper treatment. We investigated how tidal volume and oxygen delivery would contribute independently or synergistically to ventilator-induced lung injury (VILI).

**Methods:**

Under general anesthesia and tracheal intubation, healthy female C57BL/6 N mice (9 weeks old) were randomly ventilated for 2 h by standard (7 ml/kg) or high (14 ml/kg) tidal volume at positive end-expiratory pressure (PEEP) of 2 cmH_2_O, with room air, 50% O_2_ (moderate hyperoxia), or 100% O_2_ (severe hyperoxia); respectively. Mice were sacrificed 4 h after mechanical ventilation, and lung tissues were collected for experimental assessments on lung injury.

**Results:**

Compared with the healthy control, severe hyperoxia ventilation by either standard or high tidal volume resulted in significantly higher wet-to-dry lung weight ratio and higher levels of IL-1β and 8-OHdG in the lungs. However, moderate hyperoxia ventilation, even by high tidal volume did not significantly increase the levels of IL-1β and 8-OHdG in the lungs. Western blot analysis showed that the expression of RhoA, ROCK1, MLC2, and p-MLC2 was not significantly induced in the ventilated lungs, even by high tidal volume at 2 cmH_2_O PEEP.

**Conclusion:**

Severe hyperoxia ventilation causes inflammatory response and oxidative damage in mechanically ventilated lungs, while high tidal volume ventilation at a reasonable PEEP possibly does not cause VILI.

**Supplementary Information:**

The online version contains supplementary material available at 10.1186/s12890-023-02626-x.

## Introduction

Every year over 800,000 critically ill patients require mechanical ventilation in the United States [1]. Although mechanical ventilation provides essential life support, can also induce or aggravate lung injury by causing ventilator-induced lung injury (VILI) [2]. Mechanical ventilation strategies for reducing VILI in patients with apparent pulmonary diseases are well known: low tidal volume to limit overdistention, optimal oxygen level to prevent hyperoxia, and high positive end-expiratory pressure (PEEP) to prevent injury from low lung volume (atelectrauma) and alveolar collapse [3–5]. Mechanical ventilation is also often applied to patients with shock [6, 7], general anesthesia cases [8], respiratory arrest [9], and so on. Previous studies have attempted to optimize tidal volume and oxygen level to alleviate VILI in patients without apparent pulmonary diseases [3, 10–12]. However, the optimal mechanical ventilation strategy for cases without apparent pulmonary diseases is still uncertain.

Excessive high tidal volume ventilation can result in oxidative stress-induced damage, recruitment of neutrophils, and local release of inflammatory mediators in lungs [13, 14]. VILI involves direct tissue damage due to high mechanical stretch and indirect tissue damage by transducing mechanical stress to activate specific intracellular pathways involved in “mechanotransduction” in lung cells. Previous studies have demonstrated the critical role of mechanotransduction signaling pathways, mainly including Rho-associated protein kinase (ROCK) signaling pathway in VILI. Rho GTPases are signaling G proteins that are distributed across the lower surface of the cell and regulate cytoskeletal dynamics by controlling actin polymerization and myosin II-mediated contraction [15, 16].

Oxygen administration is important for preventing or correcting hypoxemia. Exposure to hyperoxia is a recognized cause of lung injury, producing histopathologic changes similar to those seen in VILI, including oxidative stress and inflammatory response [17–19]. Oxidative damage is mediated by reactive oxygen species (free radicals) derived directly from molecular oxygen. The accumulation of free hydroxyl radicals and peroxynitrite results in the oxidation of proteins and peroxidation of membrane lipids and nucleic acids [20, 21]. Despite the near-ubiquitous concomitant use of mechanical ventilation and oxygen delivery, little is known about the independent and synergistic effects of tidal volume and oxygen delivery on VILI underlying healthy lungs.

In this study, we purposed to investigate how tidal volume and oxygen delivery would independently or synergistically contribute to VILI, mainly focusing on the inflammatory response and oxidative damage in the lungs.

## Materials and methods

### Animals

Nine weeks old female C57BL/6 N mice (19-22 g, CLEA, Japan) were used for the study. Mice were housed in a pathogen-free room with a controlled environment under a 12-h light-dark cycle and maintained on laboratory chow, with free access to food and water as previously described [22]. This study was approved by the Institutional Animal Care and Use Committee of Nagasaki University (No.1608251335-12). All animal procedures were performed in accordance with institutional and national guidelines.

### Mechanical ventilation protocol

**Fig. 1:**
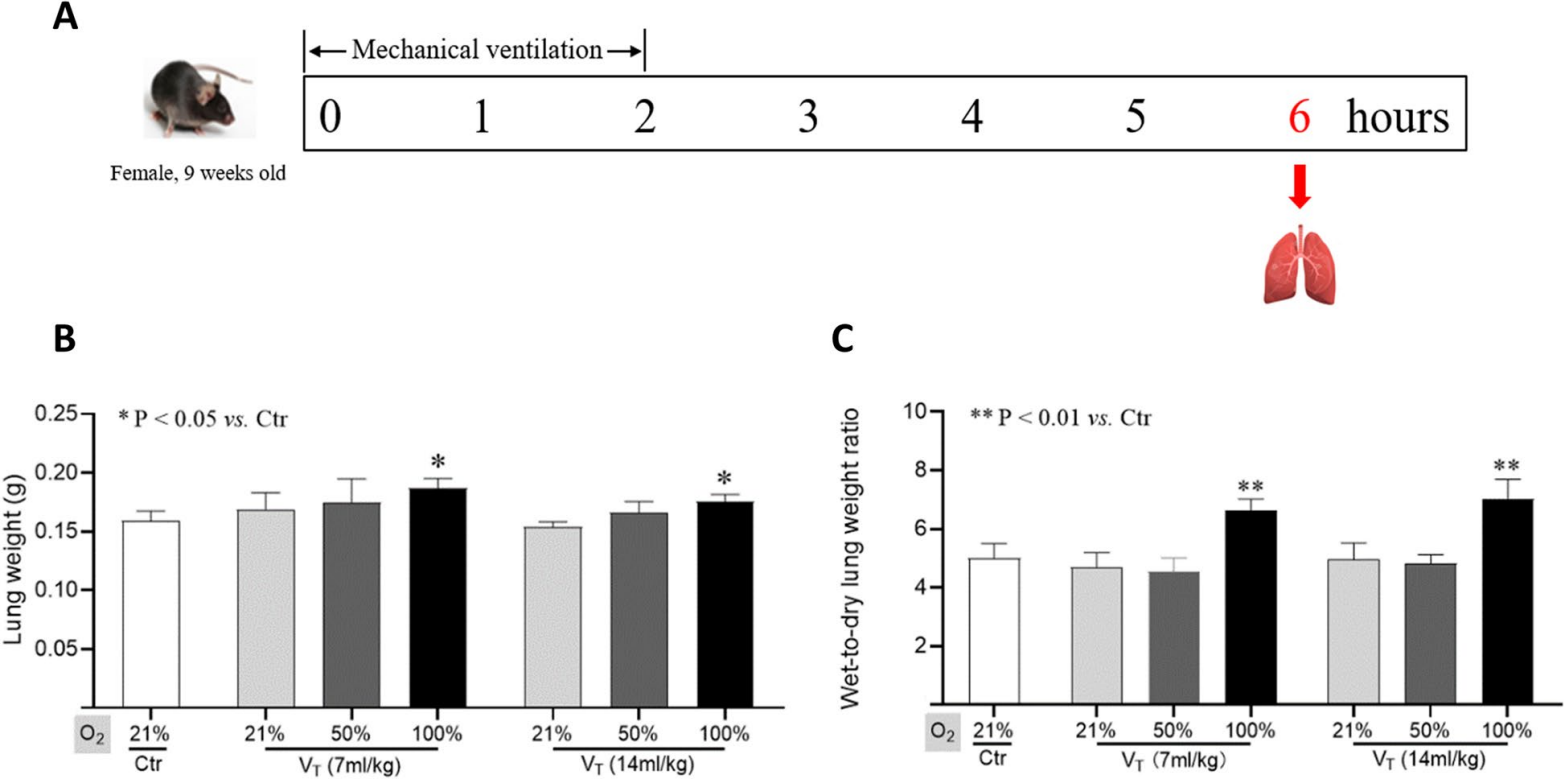
Lung weight and wet-to-dry lung weight ratio. **A**. Schematic diagram about the experimental timeline. Lung weight (**B**) and wet-to-dry lung weight ratio (**C**) are shown. Data are represented as the means ± SD, *n* = 3 ~ 5 in each group. *p < 0.05, **p < 0.01 vs. Ctr group. V_T_: tidal volume

Mice were anesthetized with intraperitoneal injection of domitor (0.75 mg/kg), midazolam (4 mg/kg), vetorphale (5 mg/kg), and then orotracheally intubated with a 20 g intravenous indwelling catheter and attached to a mini ventilator (MiniVent Type 845, Harvard Apparatus, USA). Mice were randomized to be ventilated by standard tidal volume (7 ml/kg) [23, 24] or high tidal volume (14 ml/kg); respectively with room air (21% O_2_), 50% O_2_ (FIO_2_ = 0.5, moderate hyperoxia), or 100% O_2_ (FIO_2_ = 1.0, severe hyperoxia) for 2 h (Fig. 1A). The ventilation rate was 120 breaths/min. We used 2 cmH_2_O PEEP in the official experiments, but high tidal volume ventilation with room air (21% O_2_) at 10 cm and 20 cmH_2_O PEEP were also tested for inducing lung injury. Non-ventilated mice under general anesthesia with spontaneous breathing were used as controls and were kept on spontaneous breathing for 6 h before being sacrificed. Body temperature was maintained using a 37 °C heating pad.

### Tissue sampling

Mice were sacrificed at 4 h after mechanical ventilation (Fig. 1A). After flushing with PBS via right ventricle to remove the blood, the lungs were extracted and weighed. The left lobe was fixed with 4% paraformaldehyde for paraffin sections. The right superior lobe was used for measuring the wet-to-dry lung weight ratio. The other lobes of the right lung tissue samples were stored under − 80˚C, and used for other experimental analyses.

### Lung wet-to-dry weight ratio

The wet-to-dry lung weight ratio is widely used as a parameter for the assessment of pulmonary edema. The freshly extracted lung tissue sample of the right superior lobe was weighed and recorded as the wet weight. After the incubation in an oven at 80 °C for 24 h, the dried tissue sample was weighed and recorded as dry weight. The wet-to-dry lung weight ratio was calculated.

### Immunohistochemical analysis

The oxidative injury of the lungs was detected by immunohistochemical analysis as previously described [22]. Briefly, paraffin sections of 5-µm-thick were deparaffinized and rehydrated. After antigen retrieval and blocking, sections were incubated with anti-mouse 8-OHdG antibody (1:100 dilution, Santa Cruz) overnight at 4℃, and followed by the appropriate fluorescent-conjugated secondary antibodies at 25 °C for 60 min. The nuclei were stained with 4, 6-diamidino-2-phenylindole (DAPI) (1:1000 dilution, Life technologies). The positive staining was examined under fluorescence microscope (FV10CW3, OLYMPUS). The percentage of 8-OHdG-positive cells was calculated from 12 randomly selected fields of view (6 fields/slide in 2 slides) and used for statistical analysis.

### ELISA

To evaluate the inflammatory response, ELISA kits (R&D Systems) were used to detect the contents of transforming growth factor β1 (TGF-β1) and interleukin-1beta (IL-1β) in lung tissue lysates as previously described [25]. Briefly, the lung tissues were homogenized using Multi-beads shocker® and added to the T-PER reagent (Thermo Fisher Scientific) consisting of proteinase and dephosphorylation inhibitors (Thermo Fisher Scientific). Lung tissue lysates (100 µg protein) were added to each well and measured following the manufacturer’s instructions. The optical density was measured at 450 nm using a microplate reader (Multiskan Fc, Thermo Fisher Scientific).

### RT-qPCR analysis

RT-qPCR was performed to evaluate the gene expression of *Rhoa, Rock1, Rock2, Tgfb1* and *Actb*. Briefly, total RNA was isolated from the lung tissues using Quick-RNATMMicroPrep Kit (Zymo Research, Irvine, CA, USA). RNA concentration and purity were measured by a NanoDrop2000 spectrophotometer (Thermo-Fisher Scientific, Wal-tham, MA, USA) and 1.25 µg RNA was reverse‐transcribed using the SuperScript™ VILO™ cDNA Synthesis Kit (Thermo‐Fisher Scientific). Quantitative PCR was carried out with the SYBR Green real‐time PCR Master Mix (Toyobo, Osaka, Japan). The reactions were performed on a CFX96TM real‐time PCR System (Bio‐Rad). The gene expression was normalized by housekeeping gene *Actb*. Primers were the following: *Rhoa* (Forward: 5’- TGCTTGCTCATAGTCTTCA-3’, Reverse:5’-CCAACTCTACCTGCTTCC-3’); *Rock1* (Forward: 5’- AGC TTT TGTTGG CAA TCA GC -3’, Reverse:5’- ACT TTC CTGCAA GCT TTT ATC CA -3’); *Rock2* (Forward: 5’- CAGTCC CTG GGT AGT TCA GC -3’, Reverse:5’- GCCTGG CAT ATACTCCATC-3’); *Tgfb1* (Forward:5’-ATTCCTGGCGTTACCTTG-3’, Reverse:5’-CTGTATTCCGTCTCCTTGG-3’); *Actb* (Forward:5’-GCACCACACCTTCTACAA-3’, Reverse:5’-TACGACCAGAGGCATACA-3’).

### Western blot

Western blot was performed as previously described [22]. Total proteins (30 ug) from lung tissue were separated by SDS-PAGE gels and then transferred to 0.22-µm PVDF membranes (Bio-Rad). After blocking, the membranes were incubated with primary antibodies against RhoA (1:1000 dilution; cat. no. 2117s; CST), ROCK1 (1:1000 dilution; ab156284; Abcam), MLC2 (1:1000 dilution; cat. no. 3672s; CST), p-MLC2 (1:1000 dilution; cat. no. 3671s; CST), 8-OHdG (1:500 dilution; sc-393,871; Santa Cruz), or α-Tubulin (1:1000 dilution; cat. no. 3873 S; CST) overnight at 4˚C, respectively; followed by the appropriate horseradish peroxidase-conjugated secondary antibodies (Dako). The expression was visualized using an enhanced chemiluminescence detection kit (Thermo Scientific). Semiquantitative analysis was done using ImageQuant LAS 4000 mini (GE Healthcare Life Sciences). Additional file 1 is the original WB image in the manuscript.

### Statistical analysis

Statistical analysis was performed as previously described [25]. All the values were presented as the mean ± SD. For comparison of multiple sets of data, one-way analysis of variance (ANOVA) followed by Tukey’s test (Dr. SPSS II, Chicago, IL) was used for statistical analyses. All analyses were carried out with the SPSS19.0 statistical software (IBM SPSS Co., USA). A *p*-value less than 0.05 was accepted as significant.

## Results

### Mechanical ventilation with severe hyperoxia at 2 cmH_2_O PEEP significantly induces lung injury

All mice survived in the official experiments (Fig. 1A). Our data showed that lung weight in severe hyperoxia ventilation with standard or high tidal volume were increased significantly (*p* < 0.05 vs. control; Fig. 1B). Similarly, severe hyperoxia ventilation significantly increased the wet-to-dry lung weight ratio, regardless of standard or high tidal volume (*p* < 0.01 vs. control; Fig. 1C).

**Fig. 2:**
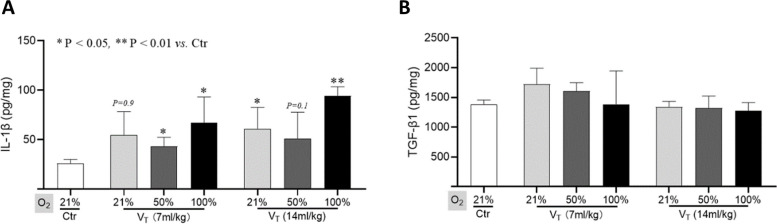
The levels of IL-1β and TGF-β1 in lung tissues. ELISA data on the levels of IL-1β (**A**) and TGF-β1 (**B**) in lungs are shown. Data are represented as the means ± SD, n = 3 ~ 5 in each group. *p < 0.05, **p < 0.01 vs. Ctr group. V_T_: tidal volume

ELISA was performed to detect the levels of IL-1β and TGF-β1. IL-1β level in the lungs was significantly increased by severe hyperoxia ventilation with either standard or high tidal volume (*p* < 0.05 or *p* < 0.01 vs. control; Fig. 2A). However, moderate hyperoxia ventilation, even by high tidal volume for 2 h did not significantly increase the IL-1β level in lungs (Fig. 2A). Consistent with previous study [26], TGF-β1 level was not significantly changed in the ventilated lungs compared with the control (Fig. 2B).

**Fig. 3:**
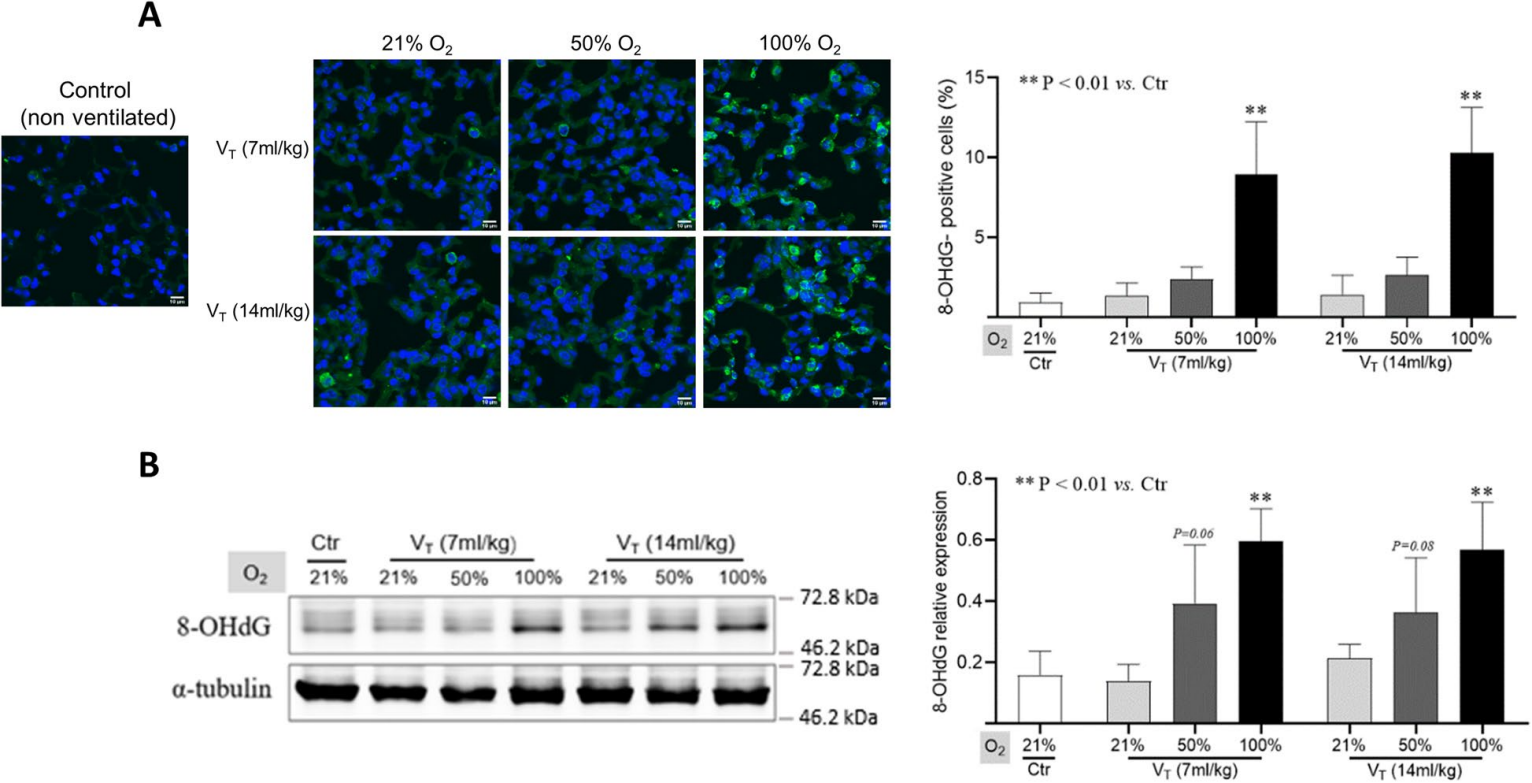
The expression of 8-OHdG in lung tissues. **A** Representative confocal images (left) and quantitative data (right) of 8-OHdG-positive cells in lung tissues. **B** Representative blots (left) and quantitative data (right) on the expression of 8-OHdG in lungs. Data are normalized to α-tubulin and represented as the means ± SD, *n* = 3 ~ 5 in each group. **p < 0.01 vs. Ctr group. V_T_: tidal volume. Additional file 1 is the original WB image in the manuscript

Oxidative stress can damage macromolecules such as DNA, lipids, and proteins, and 8-OHdG is a marker of oxidative damage to DNA and RNA [27]. Immunohistochemical analysis of lung tissues showed that severe hyperoxia, not moderate hyperoxia ventilation with high or standard tidal volume significantly increased the expression of 8-OHdG in the lungs (*p* < 0.01 vs. control; Fig. 3A). Moreover, Western blot also confirmed the enhancement of 8-OHdG in the ventilated lungs with severe hyperoxia (*p* < 0.01 vs. control; Fig. 3B).

### Relatively high tidal volume ventilation at 2 cmH_2_O PEEP does not clearly induce lung injury

Previous studies have reported that excessive high tidal volume (> 25 ml/kg) ventilation can independently lead to lung injury [4, 28]. In this study, a relatively high tidal volume (14 ml/kg, double of standard) ventilation at 2 cmH_2_O PEEP for 2 h did not significantly increase the wet-to-dry lung weights ratio, as well as the levels of IL-1β and 8-OHdG in the lungs (Figs. 2 and 3). This suggests inconspicuous damage in the lungs.

**Fig. 4:**
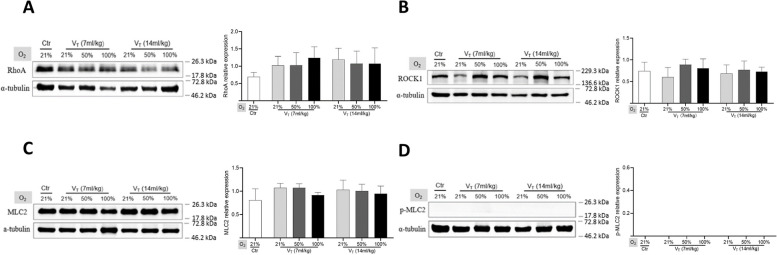
Western blot analysis on the expression of RhoA, ROCK1, MLC2, and p-MLC2 in lungs. Representative blots (left) and quantitative data (right) on the expression of RhoA (**A**), ROCK1 (**B**), MLC2 (**C**), and p-MLC2 (**D**). Data are normalized to α-tubulin and represented as the means ± SD, *n* = 3 ~ 5 in each group. V_T_: tidal volume. Additional file 1 is the original WB image in the manuscript

**Fig. 5:**
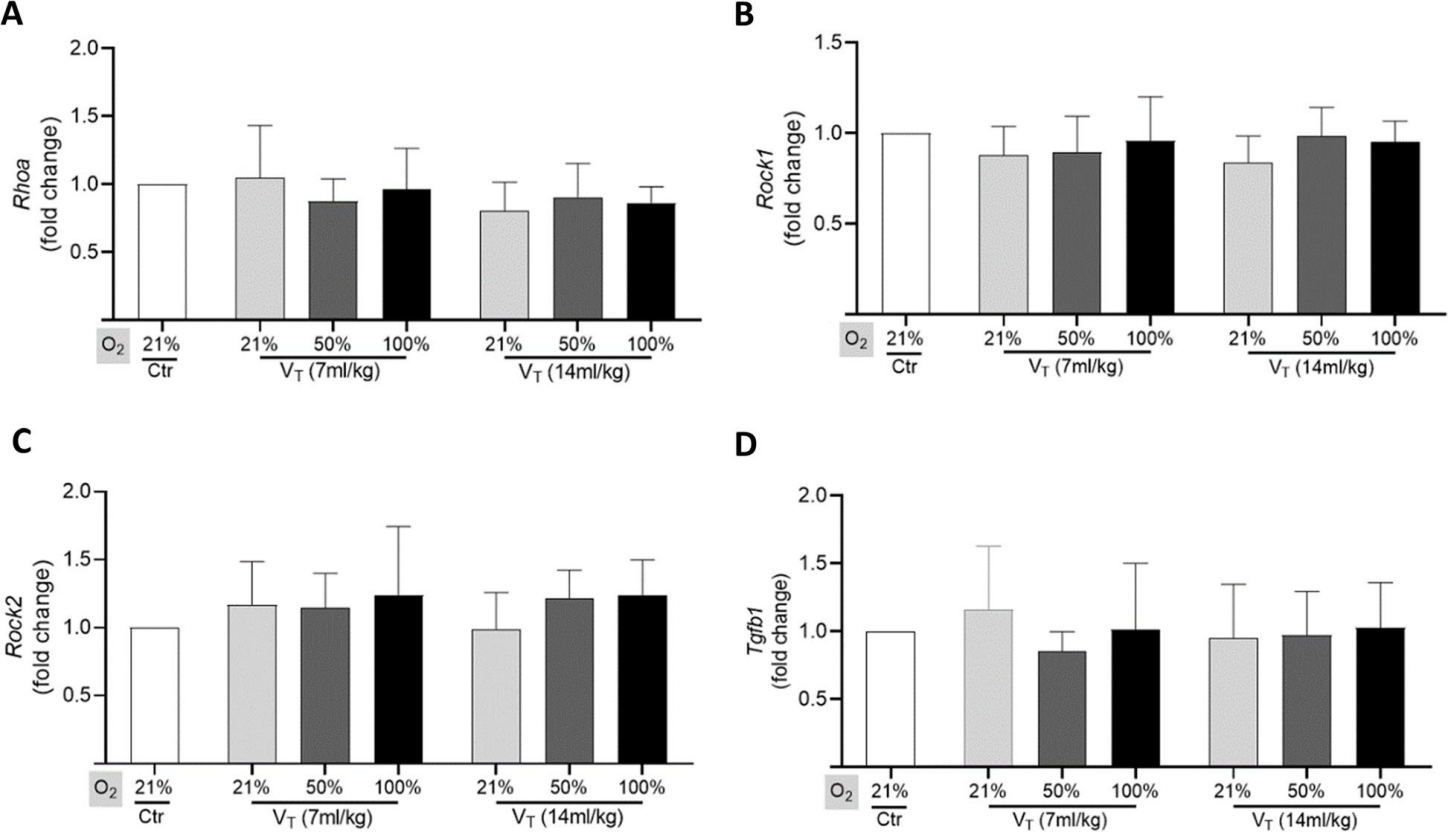
RT-PCR analysis on the expression of *Rhoa, Rock1, Rock2,* and *Tgfb1 i*n lungs. Quantitative RT-PCR data shows the relative expression of *Rhoa* (**A**), *Rock1* (**B**), *Rock2* (**C**), and *Tgfb1* (**D**) in lungs. Data are represented as the means ± SD, *n* = 3 ~ 5 in each group. V_T_: tidal volume

We further tried to evaluate whether high tidal volume ventilation induced the activation of mechanotransduction signaling in the lungs. Western blot analysis showed that the expression of RhoA, ROCK1, MLC2, and p-MLC2 was not significantly increased in the ventilated lungs even by high tidal volume at 2 cmH_2_O PEEP (Fig. 4). RT-qPCR analysis also confirmed that the expression of *Rhoa*, *Rock1*, *Rock2*, and *Tgfb1* was not significantly enhanced in the ventilated lungs (Fig. 5).

Maintaining some amount of PEEP is essential for patients receiving mechanical ventilation [29]. Because our data showed a non-injurious setting for high tidal volume ventilation at 2 cmH_2_O PEEP, we then tested whether VILI would be detectable clearly at higher PEEP. Mice were assigned to be ventilated with high tidal volume (14 ml/kg) with room air at 10 or 20 cmH_2_O PEEP for 2 h. All mice survived well during mechanical ventilation, but one mouse died at 1 h after mechanical ventilation at 20 cmH_2_O PEEP. Our data showed that high tidal volume ventilation at 10 or 20 cmH_2_O PEEP resulted in higher wet-to-dry lung weight ratio and enhanced the expression of IL-1β, TGF-β1, and 8-OHdG in the lungs (Supplementary Fig. 1). However, the expression of *Rhoa*, *Rock1*, and *Rock2* in the lungs kept stable or even slightly decreased at 4 h after high tidal volume ventilation at 10 or 20 cmH_2_O PEEP (Supplementary Fig. 2).

## Discussion

Mechanical ventilation-associated lung injury is a common clinical complication in critically ill patients. Unequivocal evidence suggests that excessive mechanical ventilation and hyperoxia have the potential to aggravate and precipitate lung injury in patients without apparent pulmonary diseases [3, 10–12]. In this study, we also found that severe hyperoxia ventilation clearly induced lung injury. In contrast, lung injury was not clearly detectable with moderate hyperoxia ventilation with a relatively high tidal volume at 2 cmH_2_O PEEP.

The levels of oxygen commonly used clinically, ranging from 50 to 100% in one atmosphere, are potentially toxic, and these patients risk exacerbation of underlying lung injury. Previous studies have demonstrated that hyperoxia augments lung injury from excessive high tidal volume ventilation in rabbits [30], in rats [31], and in ex vivo mouse lungs [32]. Although the tolerances to oxygen toxicity may be a little different between mice and human beings, healthy mice are still commonly used for experimental investigation of VILI. In this study, severe hyperoxia (100% O_2_) ventilation for 2 h significantly increased the wet-to-dry lung weight ratio, as well as IL-1β and 8-OHdG levels in the lungs, suggesting VILI. However, moderate hyperoxia (50% O_2_) ventilation did not significantly induce lung injury, even with high tidal volume. Moreover, we did not detect significant changes in TGF-β1 expression. We speculated that mechanical ventilation with relative high tidal volume (14 ml/kg) at 2 cmH_2_O PEEP for 2 h might not able to activate the TGF-β signaling pathway [26].

According to previous studies [28, 30], excessive high tidal volume (> 25ml/kg) ventilation is associated with the increased release of inflammatory cytokines and exacerbated oxidative damage. As this study is purposed to investigate VILI in patients without apparent pulmonary diseases, we only tested a relatively high tidal volume (14 ml/kg, double of standard). Based on the levels of IL-1β, TGF-β1 and 8-OHdG in the lungs, high tidal volume ventilation with air or moderate hyperoxia at 2 cmH_2_O PEEP for 2 h did not clearly induce serious injuries to the lungs. As shown in the supplemental data, to confirm that the negative data would not be a technical problem of assessment, we used several mice to test high tidal volume ventilation with room air at very high PEEP. Our data showed that high tidal volume ventilation with room air at very high PEEP (> 10 cmH_2_O) increased the levels of IL-1β, TGF-β1, and 8-OHdG in lungs, indicating VILI. Therefore, short-term ventilation with high tidal volume (14 ml/kg) at a low/physiological level of PEEP will be harmless.

In conditions of excessive mechanical overdistension, animal models have shown different signaling pathways involved in the induction of lung injury through mechanical transduction, including RhoA/ROCK signaling, and the MLC phosphorylation of downstream targets of ROCK [15, 16]. Although a relatively high tidal volume (14 ml/kg) ventilation did not cause obvious lung injury in our study, we were still interested to know about the activation of the mechanotransduction signalings in the lungs. Unexpectedly, the expression of RhoA, ROCK1, MLC2, and p-MLC2 was not significantly changed in the lungs ventilated with a relatively high tidal volume at 2 cmH_2_O PEEP. Furthermore, the expression of *Rhoa*, *Rock1*, and *Rock2* in the lungs even slightly decreased at 4 h after high tidal volume ventilation at 10 or 20 cmH_2_O PEEP, although lung injuries were clearly detectable. Several reasons can be considered about the “inactivation” of mechanotransduction signalings in lungs in our study. Firstly, it is difficult to detect changes in the expression of RhoA, ROCK, and MLC2 in lungs [33, 34]. Secondly, we collected the lung tissues at 4 h after mechanical ventilation administration, which might be not a suitable time window for sensitive detection about the changes of p-MLC2 according to previous reports [35, 36]. Thirdly, the anesthetic midazolam we used is a common central muscle relaxant, which might interfere with the activation of mechanotransduction signaling.

There are some limitations in our study. Firstly, it is necessary to include experimental groups of spontaneous breathing with high F_IO2_ for validating the oxygen toxicity to the lungs. Secondly, as this study was originally designed to investigate the synergistic effect of high tidal volume and hyperoxia in VILI, we only evaluated the inflammatory response by IL-1β and TGF-β levels. Thirdly, we were also not able to include more reliable parameters on VILI defination, such as respiratory mechanics and histological findings.

## Conclusions

Severe hyperoxia ventilation causes inflammatory response and oxidative damage in mechanically ventilated lungs, while high tidal volume ventilation at a reasonable PEEP possibly does not cause VILI.

### Supplementary Information

**Additional file 1.**

## Data Availability

The datasets used and/or analyzed during the current study are available from the corresponding author on reasonable request.
