# Supplementary material for: Hyperoxia but not high tidal volume contributes to ventilator-induced lung injury in healthy mice
Source: BMC Pulm Med. 2023 Sep 20;23:354. doi: 10.1186/s12890-023-02626-x (PMC10510264; doi:10.1186/s12890-023-02626-x)
Supplement: Supplementary file 1 — Additional file 1. [file 12890_2023_2626_MOESM1_ESM.pdf]

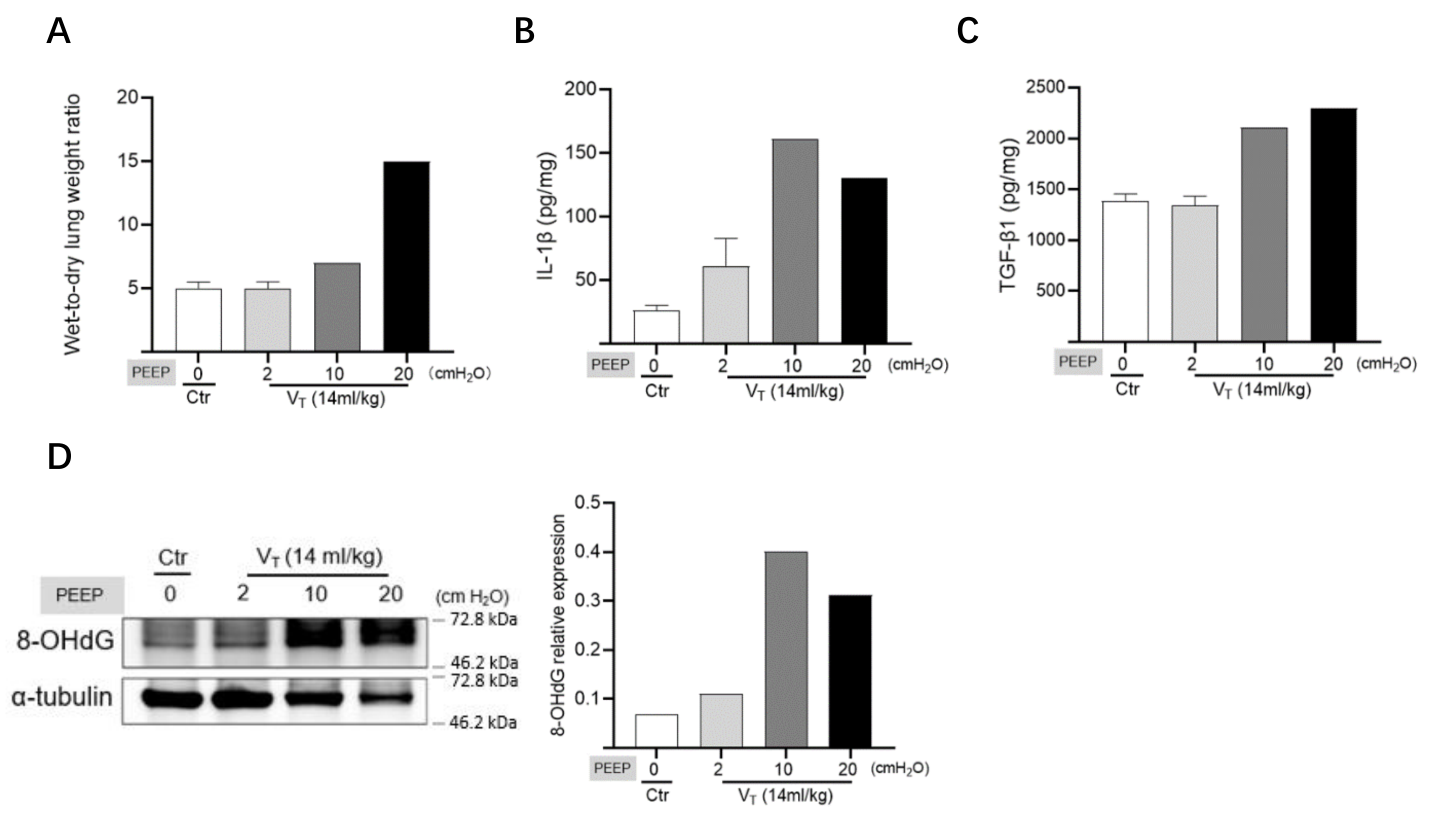

Supplementary figure 1. The assessments on lung injury in mice with high tidal volume at different PEEP.

A. The wet-to-dry lung weight ratio is shown. ELISA data on the levels of IL-1β (**B**) and TGF-β1 (**C**) in lung tissues. **D.** Representative blots (left) and quantitative data (right) on the expression of 8-OHdG in lung tissues. Data are represented as the means ± SD, n=1~5 in each group. V_T: tidal volume. Additional file 1 is the original WB image in the manuscript.

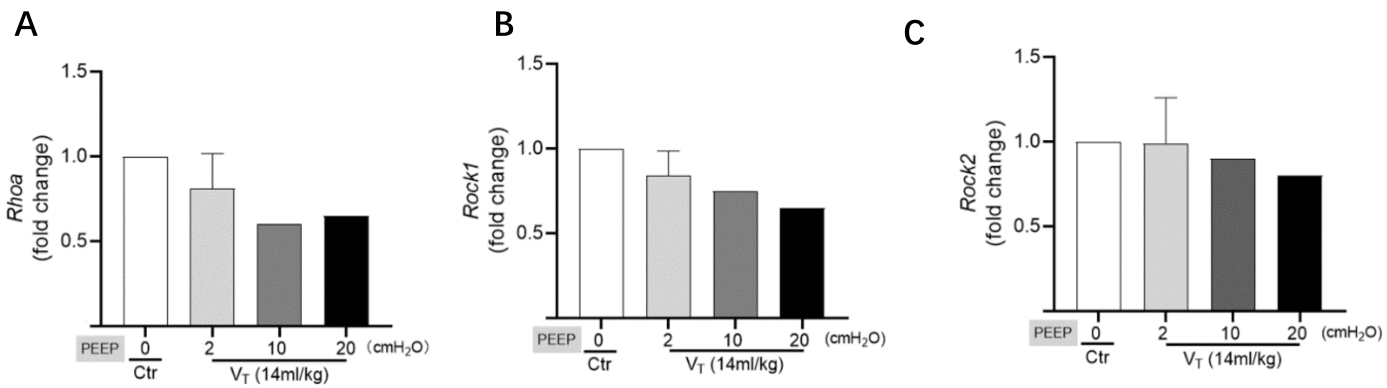

Supplementary figure 2. The expression of *Rhoa, Rock1*, *Rock2* in lungs with high tidal volume at different PEEP.

Quantitative RT-PCR data shows the relative expression of *Rhoa* (**A**), *Rock1* (**B**), *Rock2* (**C**) in lungs. Data are represented as the means ± SD, n = 1~5 in each group.

V_T: tidal volume

Supplementary Figure 3b: 8-OHdG

1

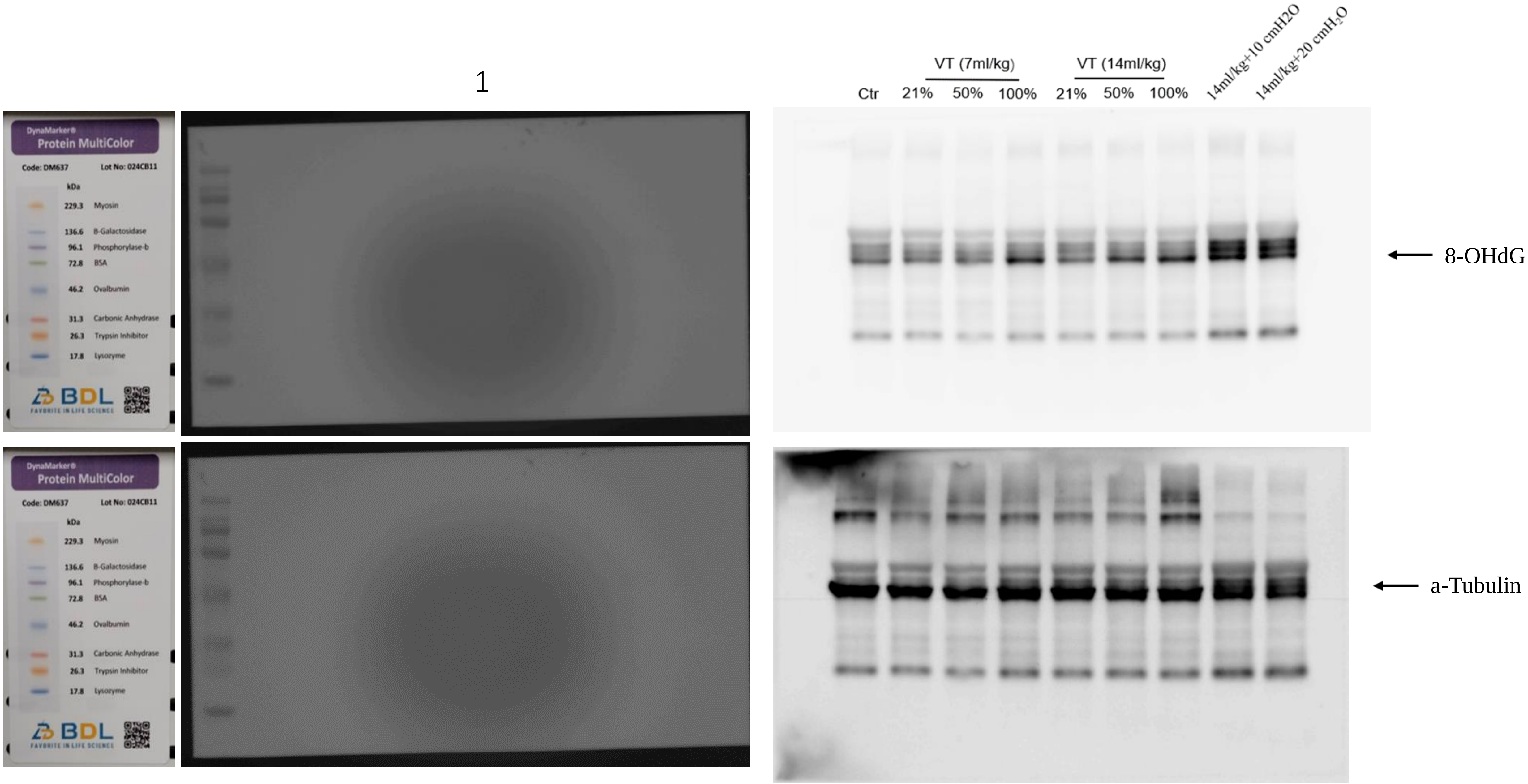
DynaMarker®
Protein MultiColor
Code: DM637
Lot No: 024CB11
kDa
229.3 Myosin
136.6 B-Galactosidase
96.1 Phosphorylase-b
72.8 BSA
46.2 Ovalbumin
31.3 Carbonic Anhydrase
26.3 Trypsin Inhibitor
17.8 Lysozyme
BDL
FAVORITE IN LIFE SCIENCE
VT (7ml/kg)
VT (14ml/kg)
Ctr
21%
50%
100%
21%
50%
100%
14ml/kg+10 cmH2O
14ml/kg+20 cmH2O
8-OHdG
a-Tubulin

2

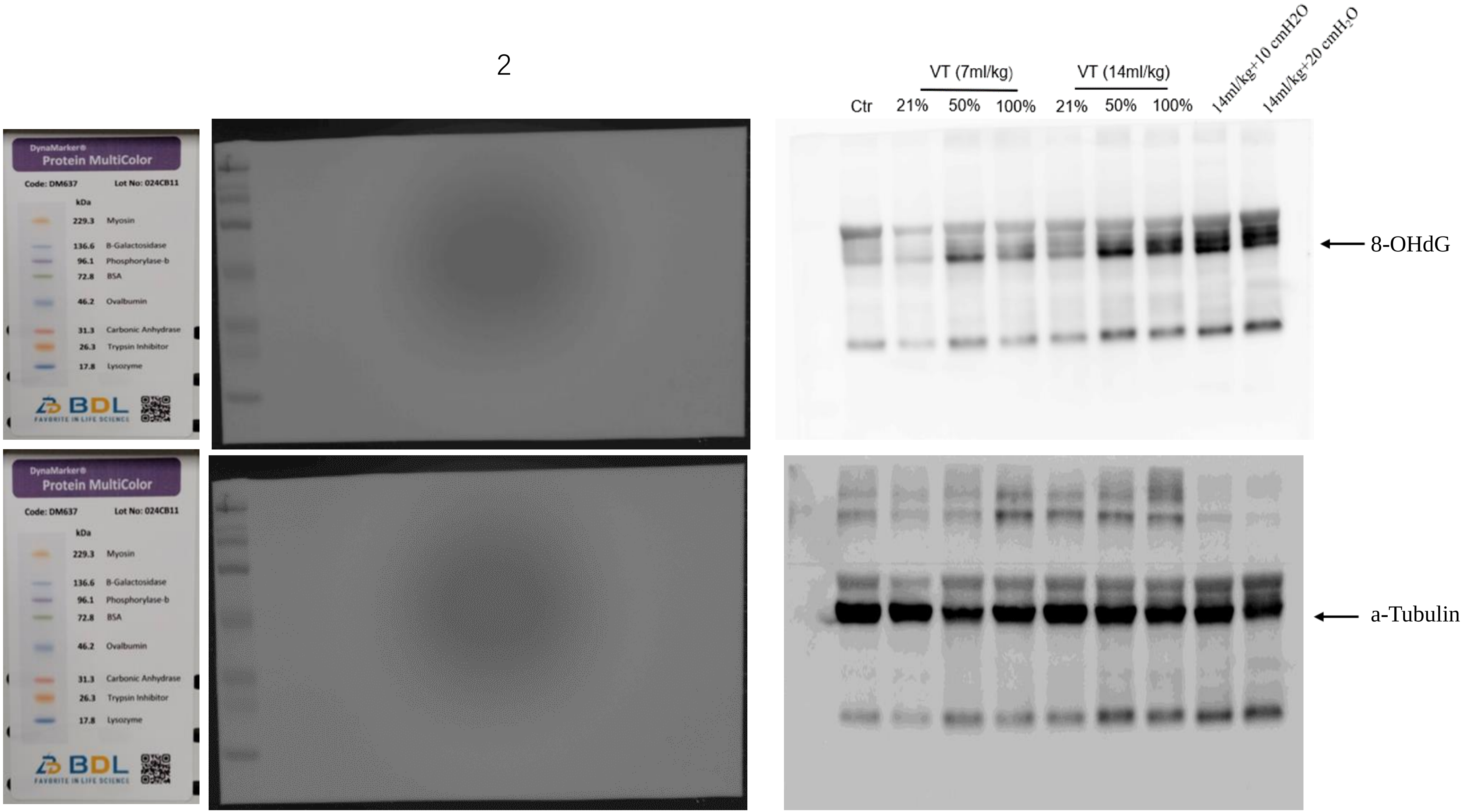
DynaMarker®
Protein MultiColor
Code: DM637
Lot No: 024CB11
kDa
229.3 Myosin
136.6 B-Galactosidase
96.1 Phosphorylase-b
72.8 BSA
46.2 Ovalbumin
31.3 Carbonic Anhydrase
26.3 Trypsin Inhibitor
17.8 Lysozyme
BDL
FAVORITE IN LIFE SCIENCE
DynaMarker®
Protein MultiColor
Code: DM637
Lot No: 024CB11
kDa
229.3 Myosin
136.6 B-Galactosidase
96.1 Phosphorylase-b
72.8 BSA
46.2 Ovalbumin
31.3 Carbonic Anhydrase
26.3 Trypsin Inhibitor
17.8 Lysozyme
BDL
FAVORITE IN LIFE SCIENCE
VT (7ml/kg)
VT (14ml/kg)
Ctr
21%
50%
100%
21%
50%
100%
14ml/kg+10 cmH2O
14ml/kg+20 cmH2O
8-OHdG
a-Tubulin

3

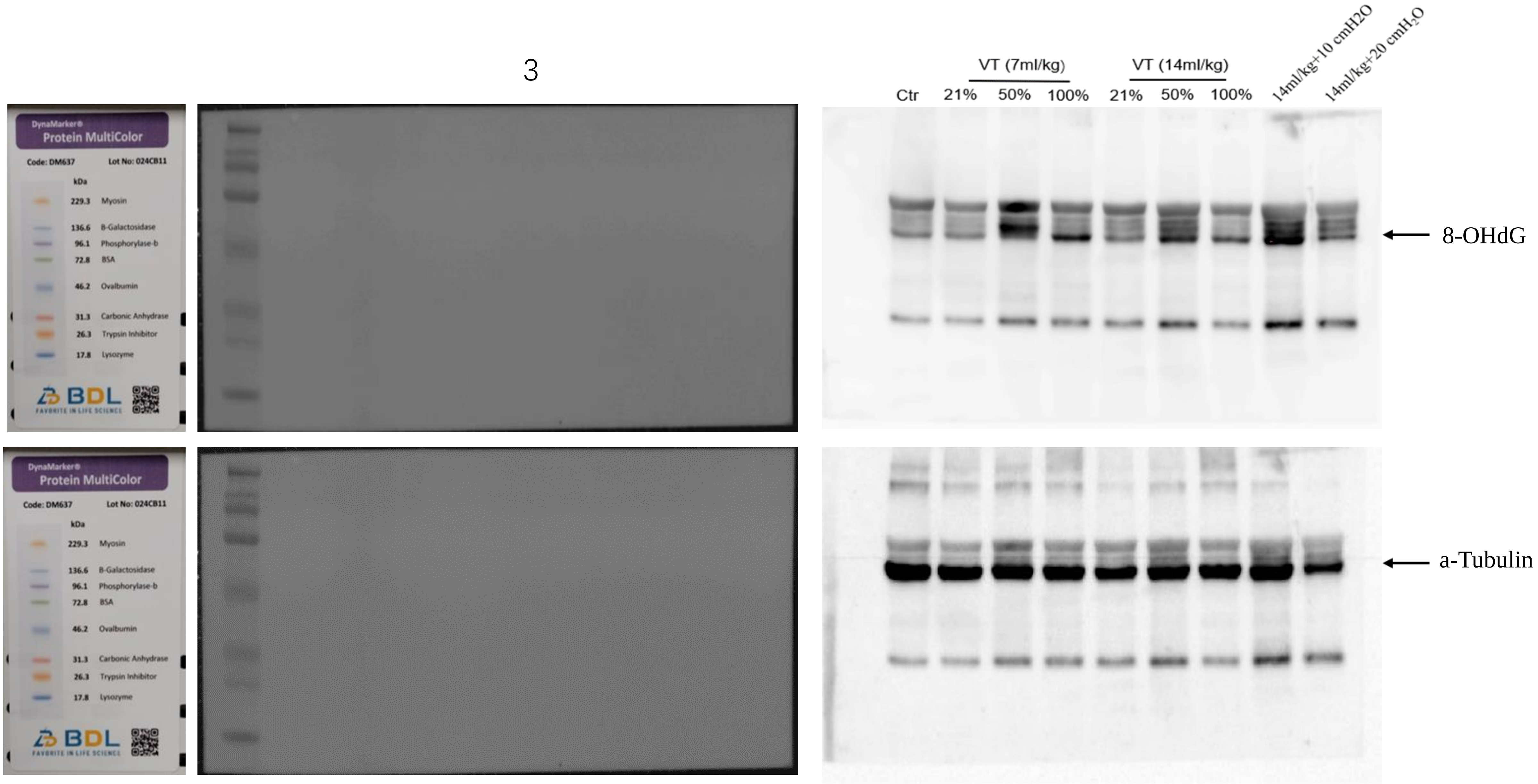
DynaMarker®
Protein MultiColor
Code: DM637
Lot No: 024CB11
kDa
229.3 Myosin
136.6 B-Galactosidase
96.1 Phosphorylase-b
72.8 BSA
46.2 Ovalbumin
31.3 Carbonic Anhydrase
26.3 Trypsin Inhibitor
17.8 Lysozyme
BDL
FAVORITE IN LIFE SCIENCE
VT (7ml/kg)
VT (14ml/kg)
Ctr
21%
50%
100%
21%
50%
100%
14ml/kg+10 cmH2O
14ml/kg+20 cmH2O
8-OHdG
a-Tubulin

4

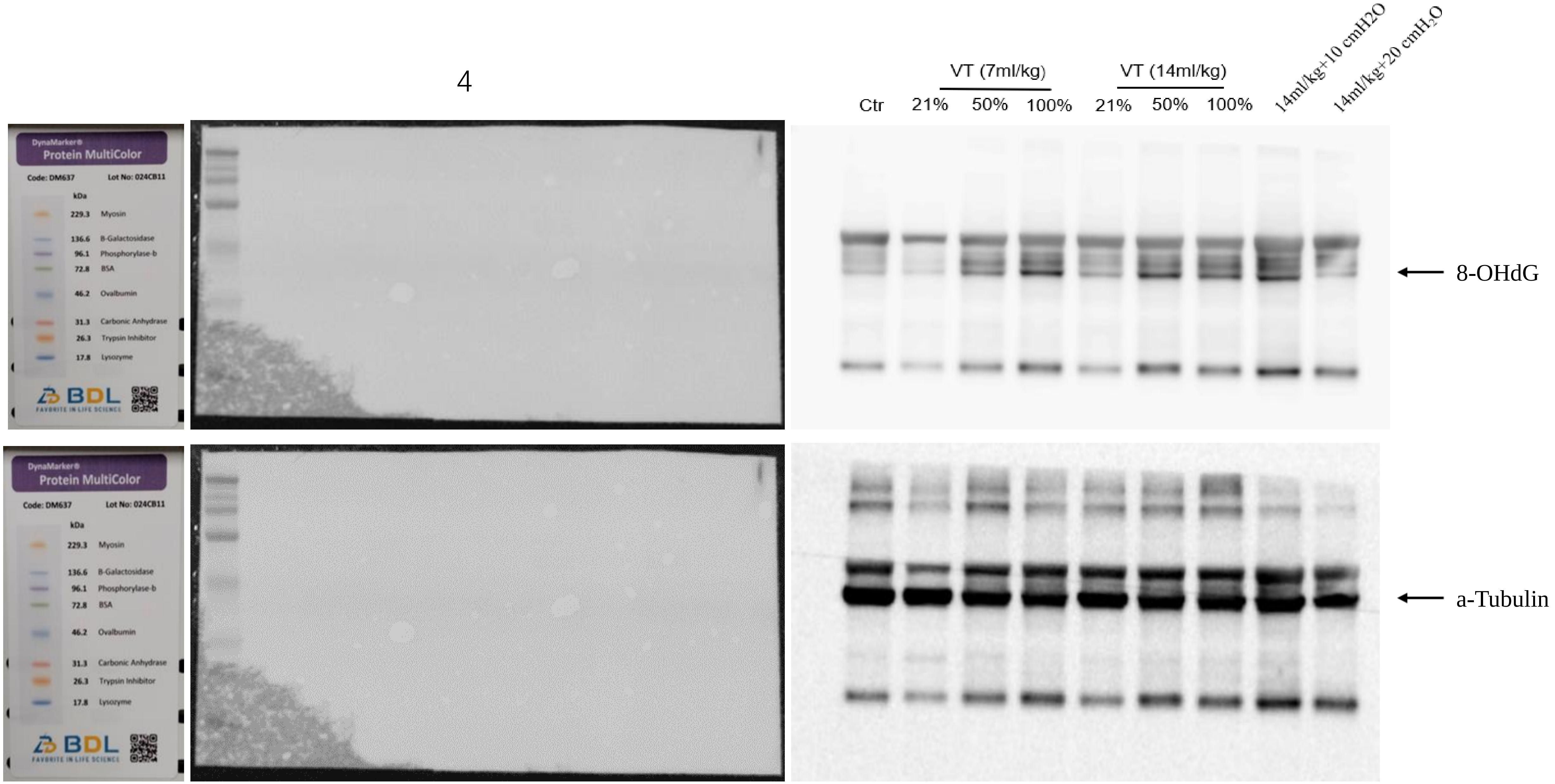
DynaMarker®
Protein MultiColor
Code: DM637
Lot No: 024CB11
kDa
229.3 Myosin
136.6 B-Galactosidase
96.1 Phosphorylase-b
72.8 BSA
46.2 Ovalbumin
31.3 Carbonic Anhydrase
26.3 Trypsin Inhibitor
17.8 Lysozyme
BDL
VT (7ml/kg)
VT (14ml/kg)
Ctr
21%
50%
100%
21%
50%
100%
14ml/kg+10 cmH2O
14ml/kg+20 cmH2O
8-OHdG
a-Tubulin

Supplementary figure 4a: RhoA

1

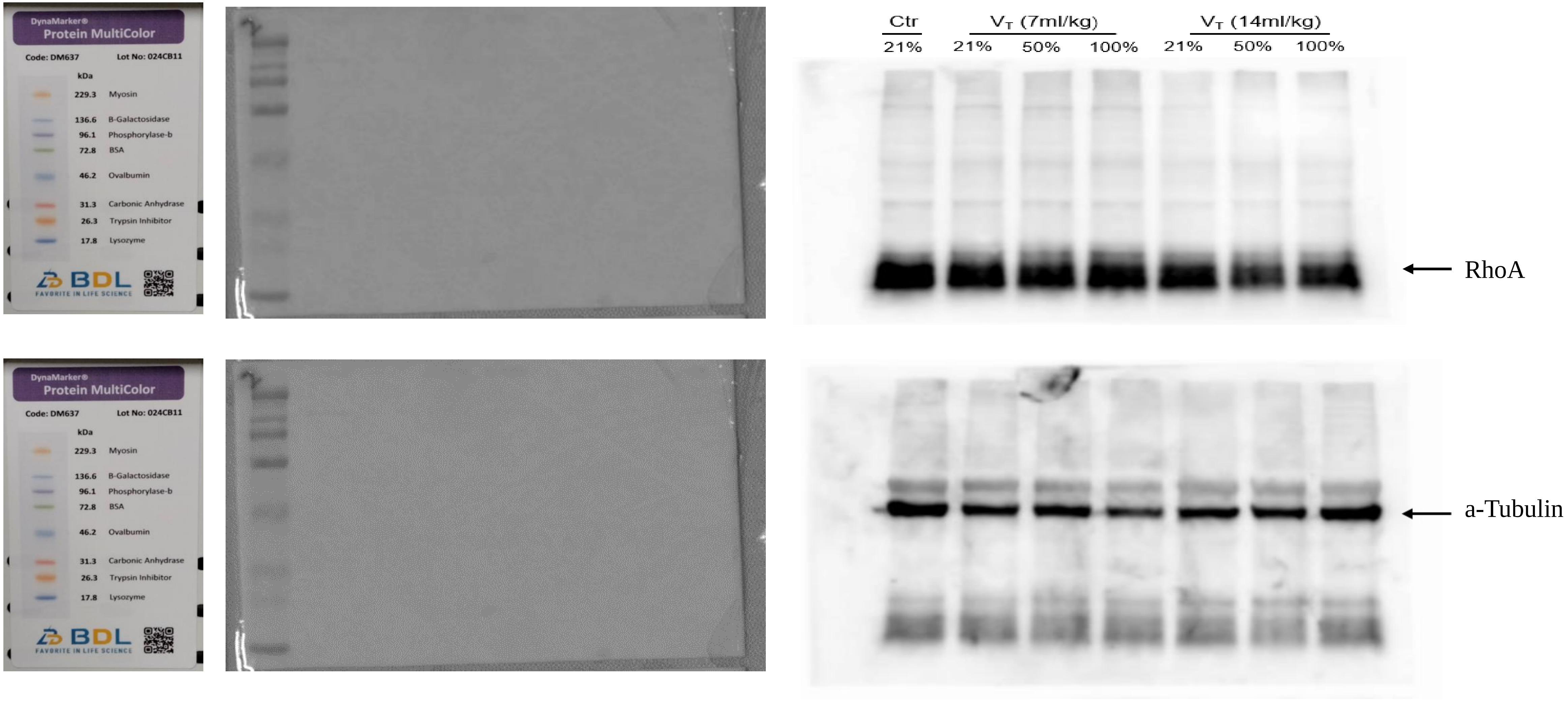
DynaMarker®
Protein MultiColor
Code: DM637
Lot No: 024CB11
kDa
229.3 Myosin
136.6 B-Galactosidase
96.1 Phosphorylase-b
72.8 BSA
46.2 Ovalbumin
31.3 Carbonic Anhydrase
26.3 Trypsin Inhibitor
17.8 Lysozyme
BDL
FAVORITE IN LIFE SCIENCE
Ctr
V_T (7ml/kg)
V_T (14ml/kg)
21%
21%
50%
100%
21%
50%
100%
RhoA
a-Tubulin

2

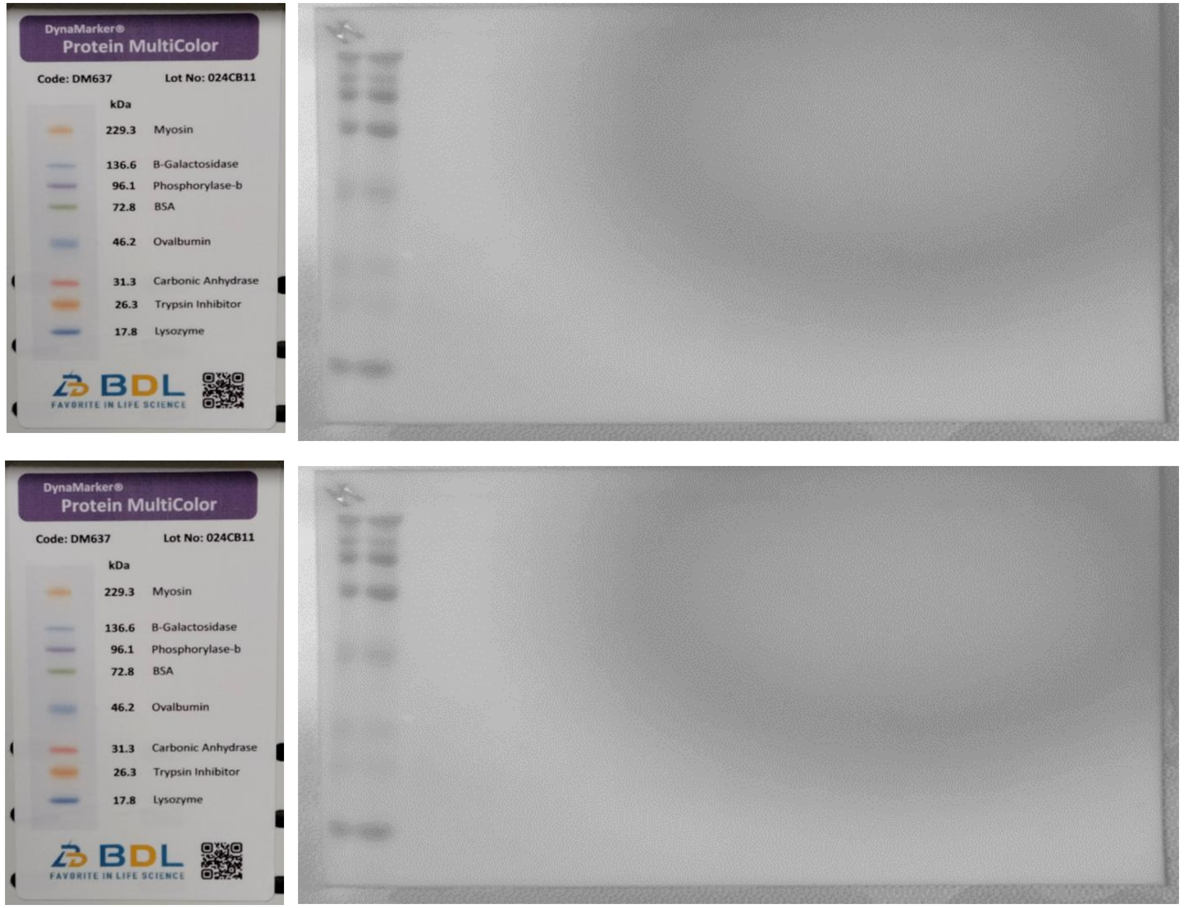
DynaMarker®
Protein MultiColor
Code: DM637
Lot No: 024CB11
kDa
229.3 Myosin
136.6 B-Galactosidase
96.1 Phosphorylase-b
72.8 BSA
46.2 Ovalbumin
31.3 Carbonic Anhydrase
26.3 Trypsin Inhibitor
17.8 Lysozyme
BDL
FAVORITE IN LIFE SCIENCE

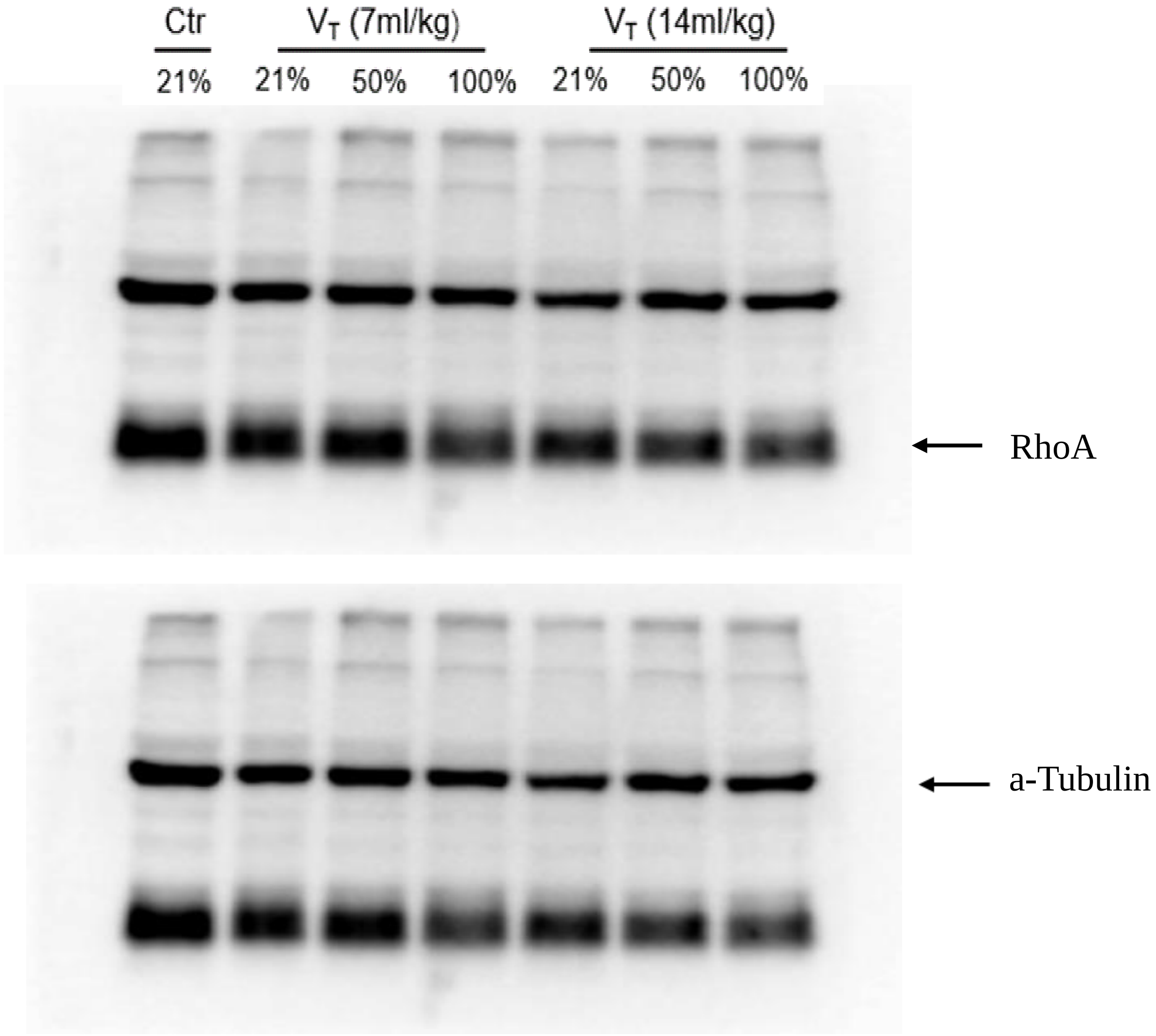
Ctr
V$_T$ (7ml/kg)
V$_T$ (14ml/kg)
21%
21%
50%
100%
21%
50%
100%
RhoA
a-Tubulin

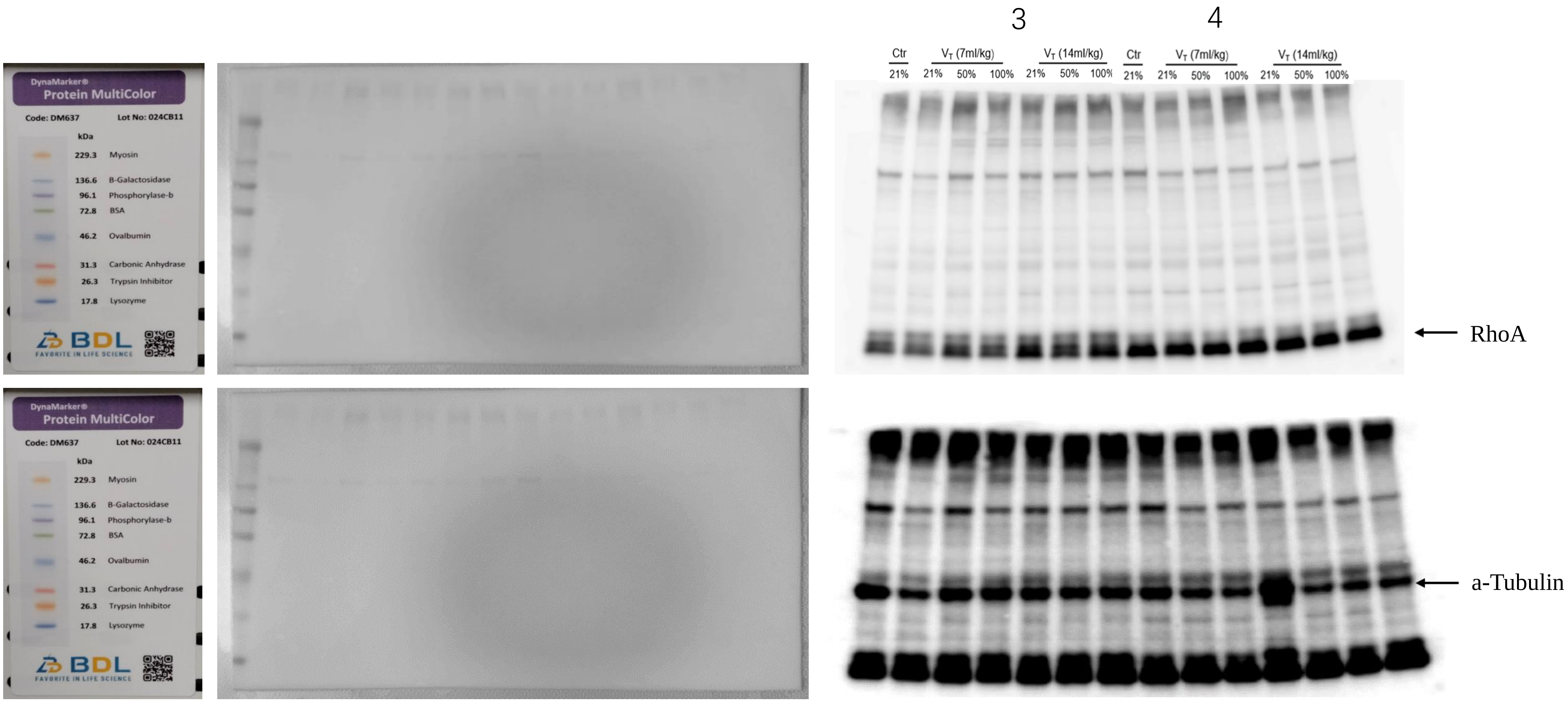
DynaMarker®
Protein MultiColor
Code: DM637
Lot No: 024CB11
kDa
229.3 Myosin
136.6 B-Galactosidase
96.1 Phosphorylase-b
72.8 BSA
46.2 Ovalbumin
31.3 Carbonic Anhydrase
26.3 Trypsin Inhibitor
17.8 Lysozyme
BDL
FAVORITE IN LIFE SCIENCE
3
4
Ctr
V_T (7ml/kg)
V_T (14ml/kg)
21% 21% 50% 100% 21% 50% 100% 21% 21% 50% 100% 21% 50% 100%
RhoA
a-Tubulin

Supplementary figure 4b: ROCK1

1

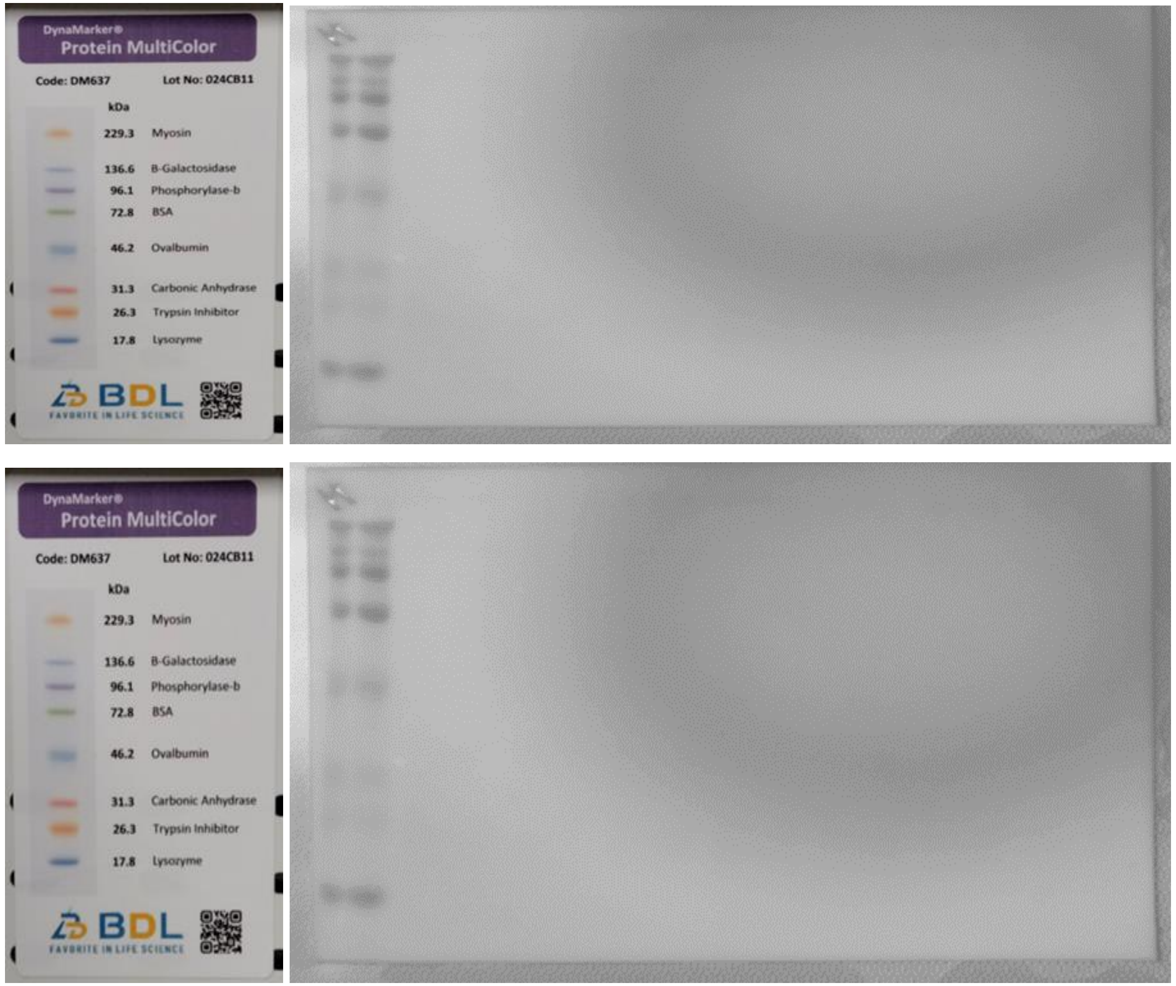
DynaMarker®
Protein MultiColor
Code: DM637
Lot No: 024CB11
kDa
229.3 Myosin
136.6 B-Galactosidase
96.1 Phosphorylase-b
72.8 BSA
46.2 Ovalbumin
31.3 Carbonic Anhydrase
26.3 Trypsin Inhibitor
17.8 Lysozyme
BDL
FAVORITE IN LIFE SCIENCE

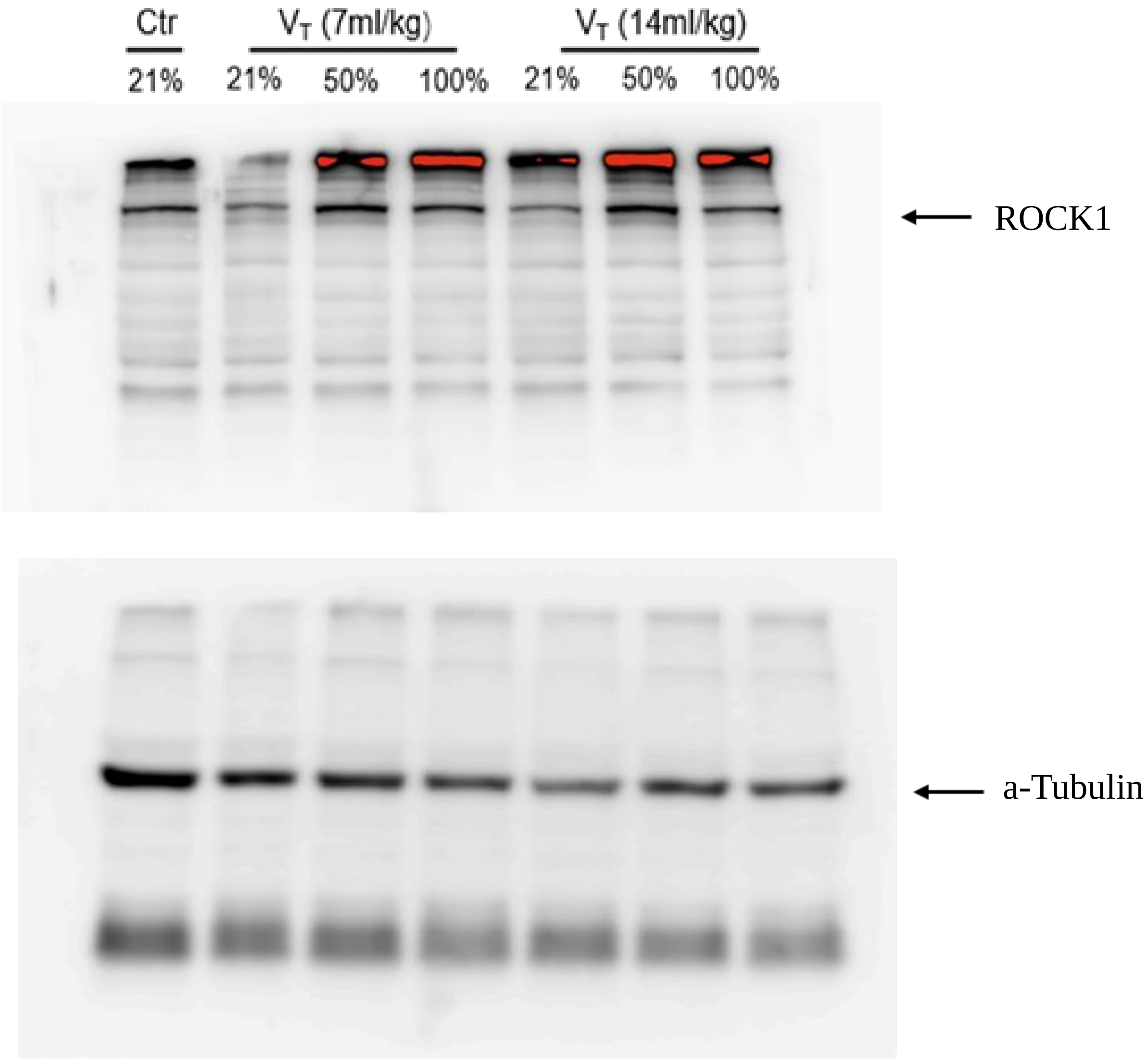
Ctr
V_T (7ml/kg)
V_T (14ml/kg)
21%
21%
50%
100%
21%
50%
100%
ROCK1
a-Tubulin

2

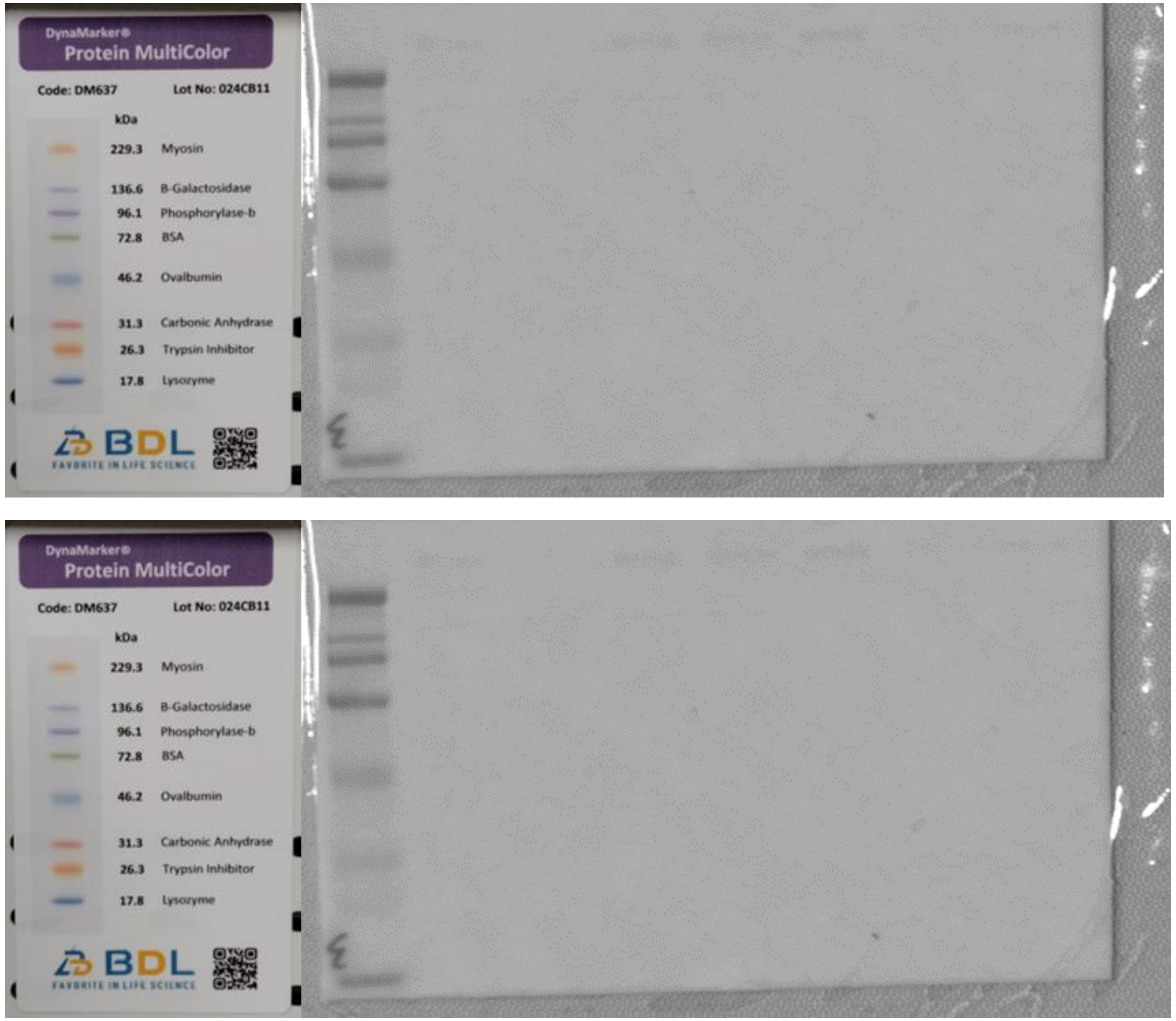
DynaMarker®
Protein MultiColor
Code: DM637
Lot No: 024CB11
kDa
229.3 Myosin
136.6 B-Galactosidase
96.1 Phosphorylase-b
72.8 BSA
46.2 Ovalbumin
31.3 Carbonic Anhydrase
26.3 Trypsin Inhibitor
17.8 Lysozyme
BDL
FAVORITE IN LIFE SCIENCE

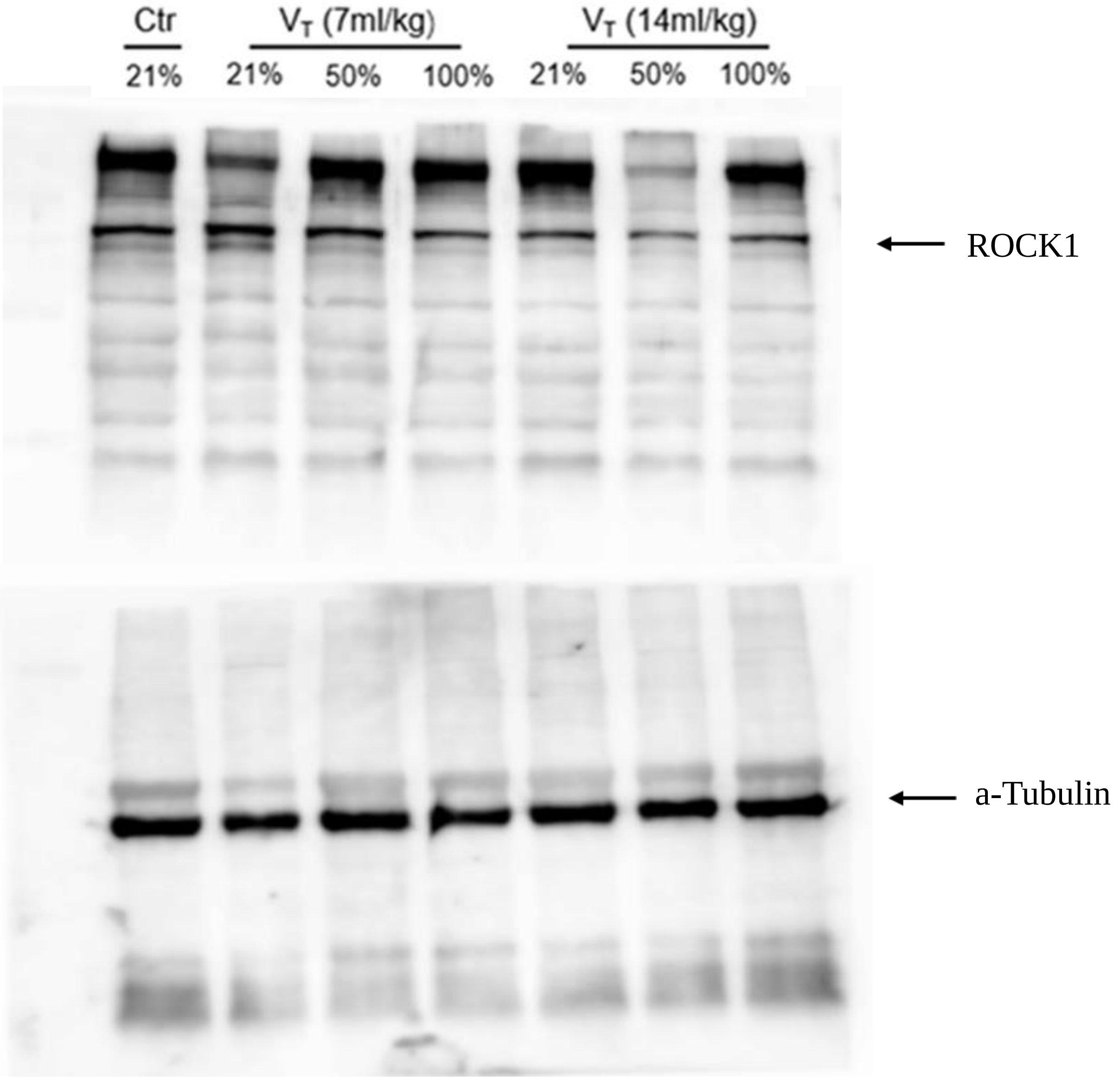
Ctr
V_T (7ml/kg)
V_T (14ml/kg)
21%
21%
50%
100%
21%
50%
100%
ROCK1
a-Tubulin

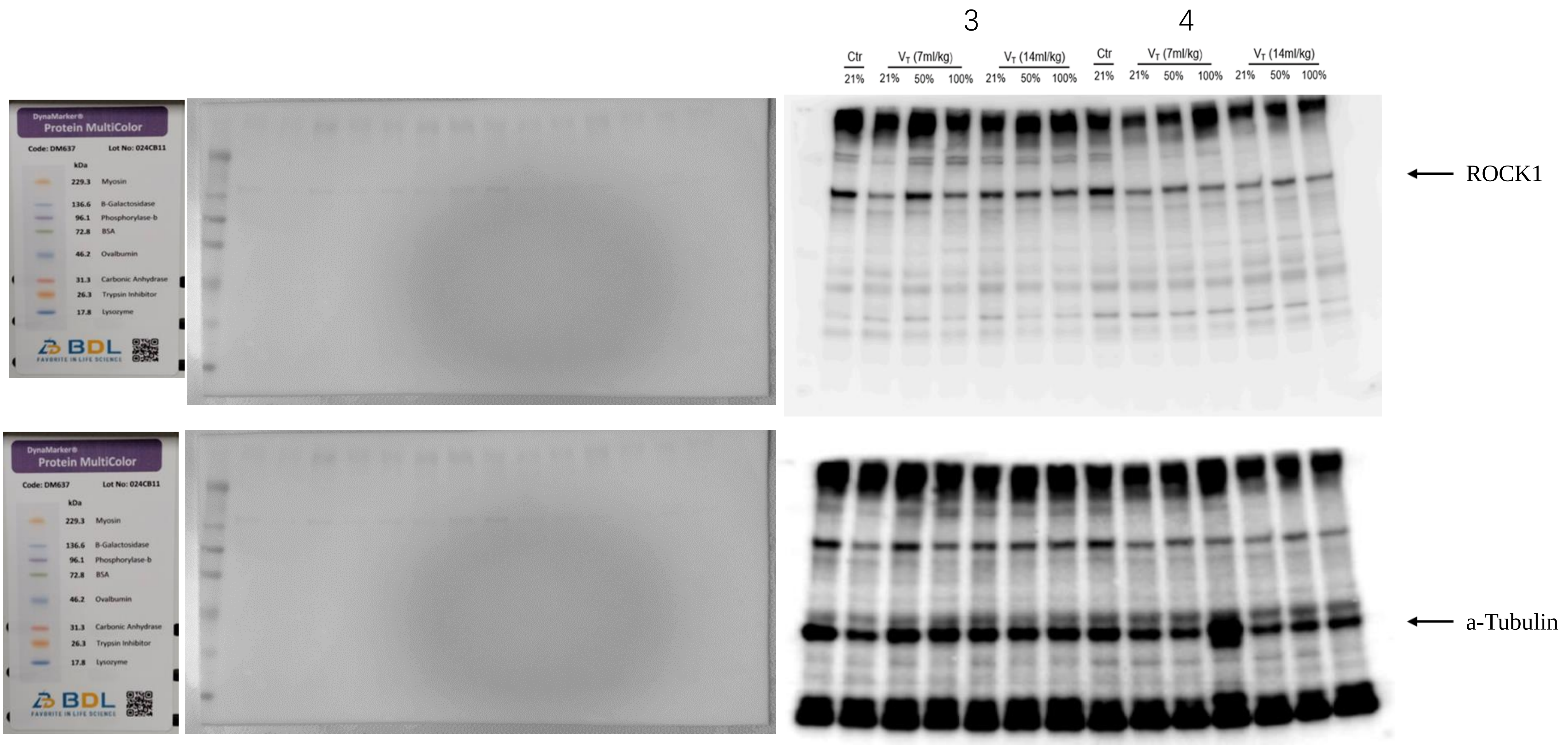

3
4
Ctr
V_T (7ml/kg)
V_T (14ml/kg)
21%
21% 50% 100%
21% 50% 100%
Ctr
V_T (7ml/kg)
V_T (14ml/kg)
21%
21% 50% 100%
21% 50% 100%
ROCK1
a-Tubulin
DynaMarker® Protein MultiColor
Code: DM637
Lot No: 024CB11
kDa
229.3 Myosin
136.6 B-Galactosidase
96.1 Phosphorylase-b
72.8 BSA
46.2 Ovalbumin
31.3 Carbonic Anhydrase
26.3 Trypsin Inhibitor
17.8 Lysozyme
BDL
FAVORITE IN LIFE SCIENCE

Supplementary figure 4c: MLC2

1

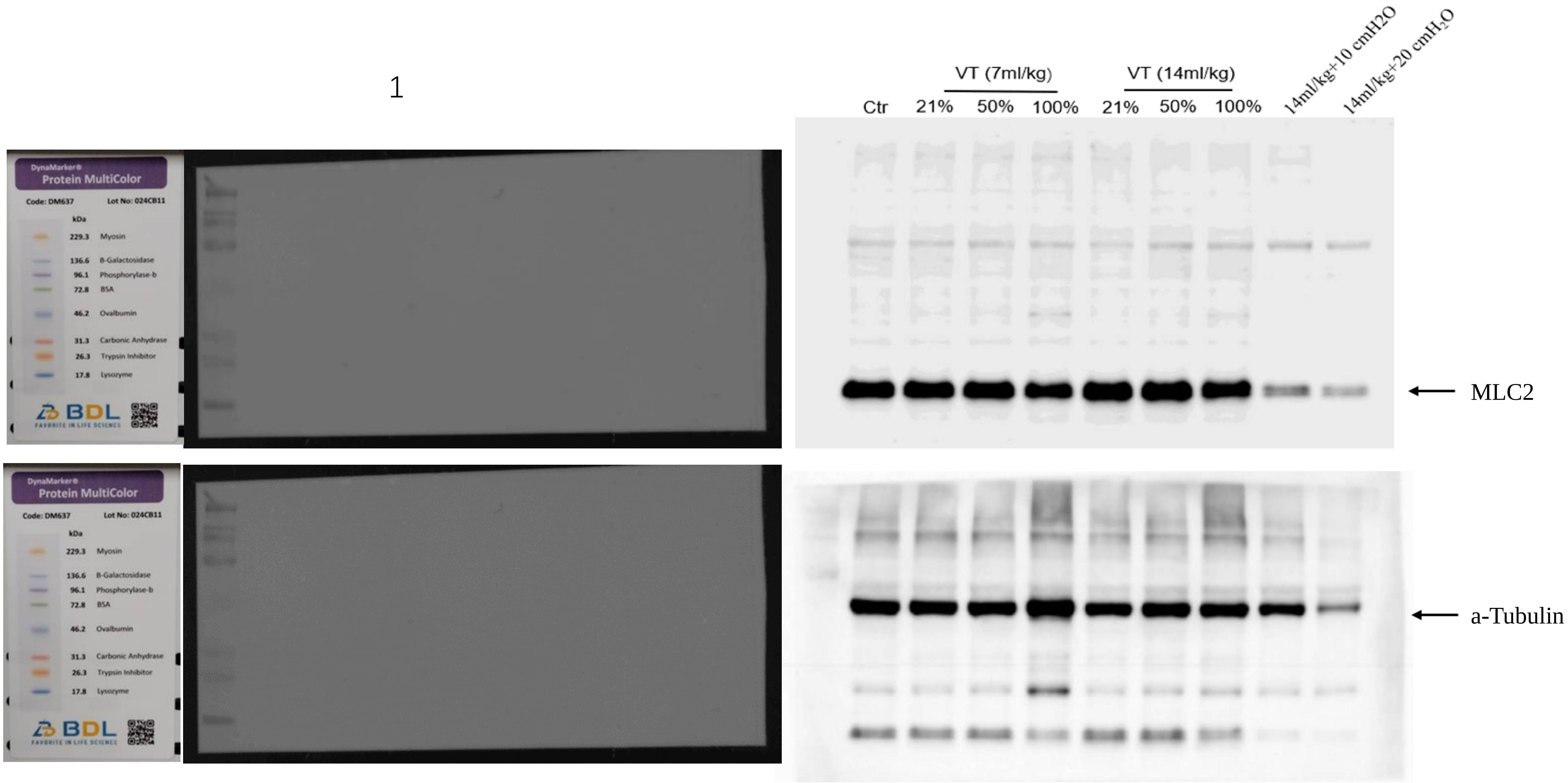
DynaMarker®
Protein MultiColor
Code: DM637
Lot No: 024CB11
kDa
229.3 Myosin
136.6 B-Galactosidase
96.1 Phosphorylase-b
72.8 BSA
46.2 Ovalbumin
31.3 Carbonic Anhydrase
26.3 Trypsin Inhibitor
17.8 Lysozyme
BDL
VT (7ml/kg)
VT (14ml/kg)
Ctr
21%
50%
100%
21%
50%
100%
14ml/kg+10 cmH2O
14ml/kg+20 cmH$_2$O
MLC2
a-Tubulin

2

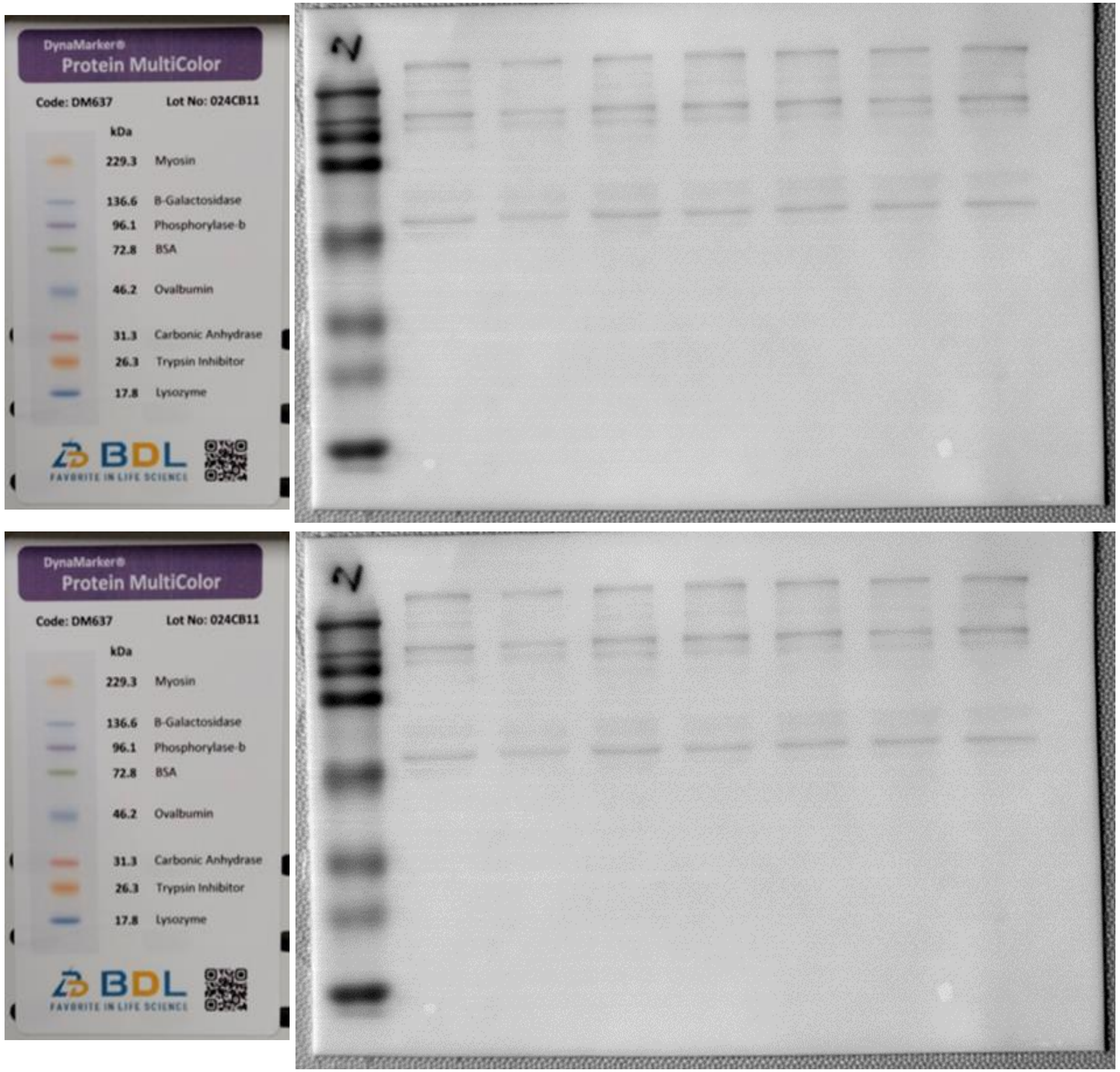
DynaMarker®
Protein MultiColor
Code: DM637
Lot No: 024CB11
kDa
229.3 Myosin
136.6 B-Galactosidase
96.1 Phosphorylase-b
72.8 BSA
46.2 Ovalbumin
31.3 Carbonic Anhydrase
26.3 Trypsin Inhibitor
17.8 Lysozyme
BDL
FAVORITE IN LIFE SCIENCE
DynaMarker®
Protein MultiColor
Code: DM637
Lot No: 024CB11
kDa
229.3 Myosin
136.6 B-Galactosidase
96.1 Phosphorylase-b
72.8 BSA
46.2 Ovalbumin
31.3 Carbonic Anhydrase
26.3 Trypsin Inhibitor
17.8 Lysozyme
BDL
FAVORITE IN LIFE SCIENCE

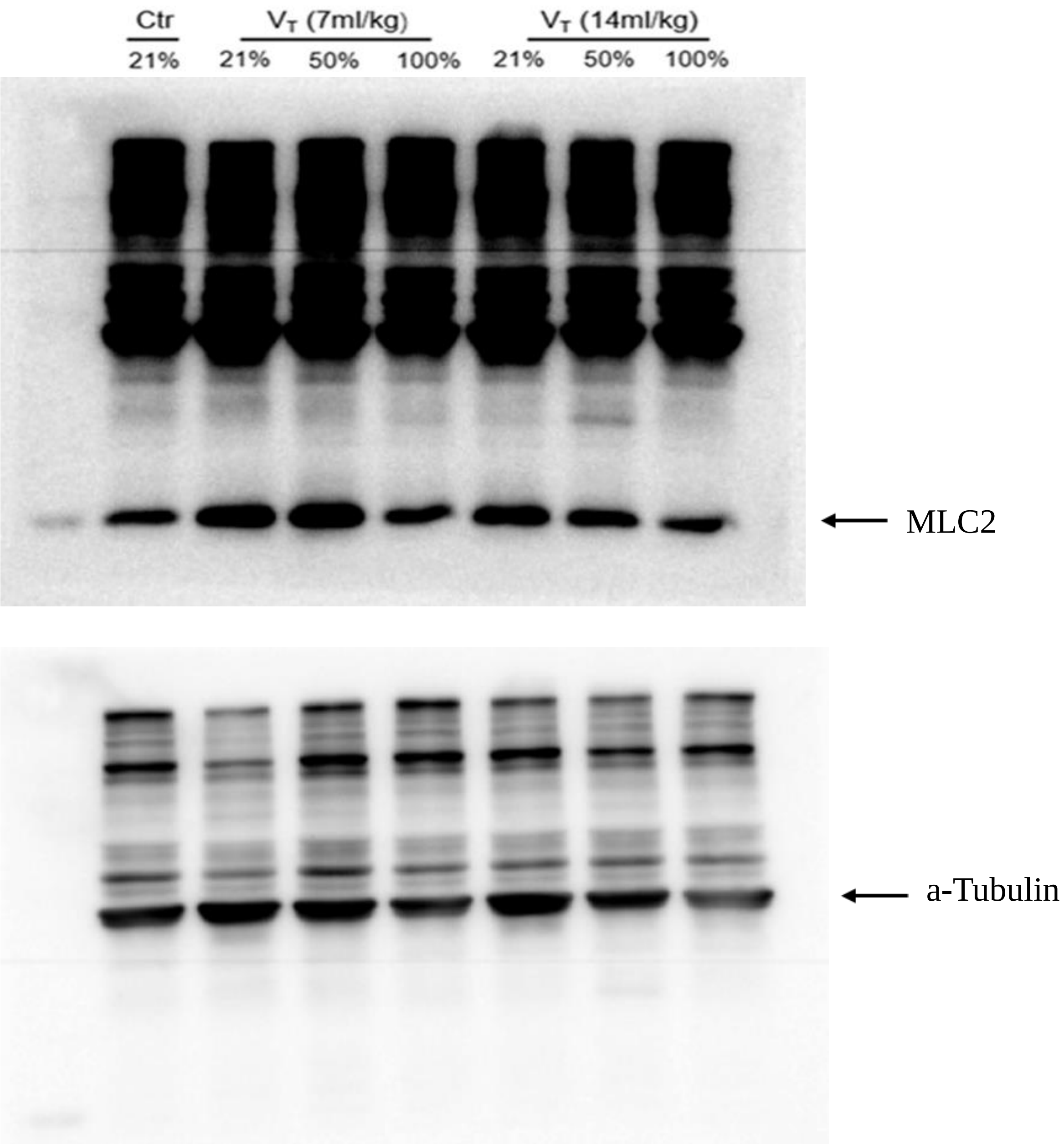
Ctr
V_T (7ml/kg)
V_T (14ml/kg)
21%
21%
50%
100%
21%
50%
100%
MLC2
a-Tubulin

3

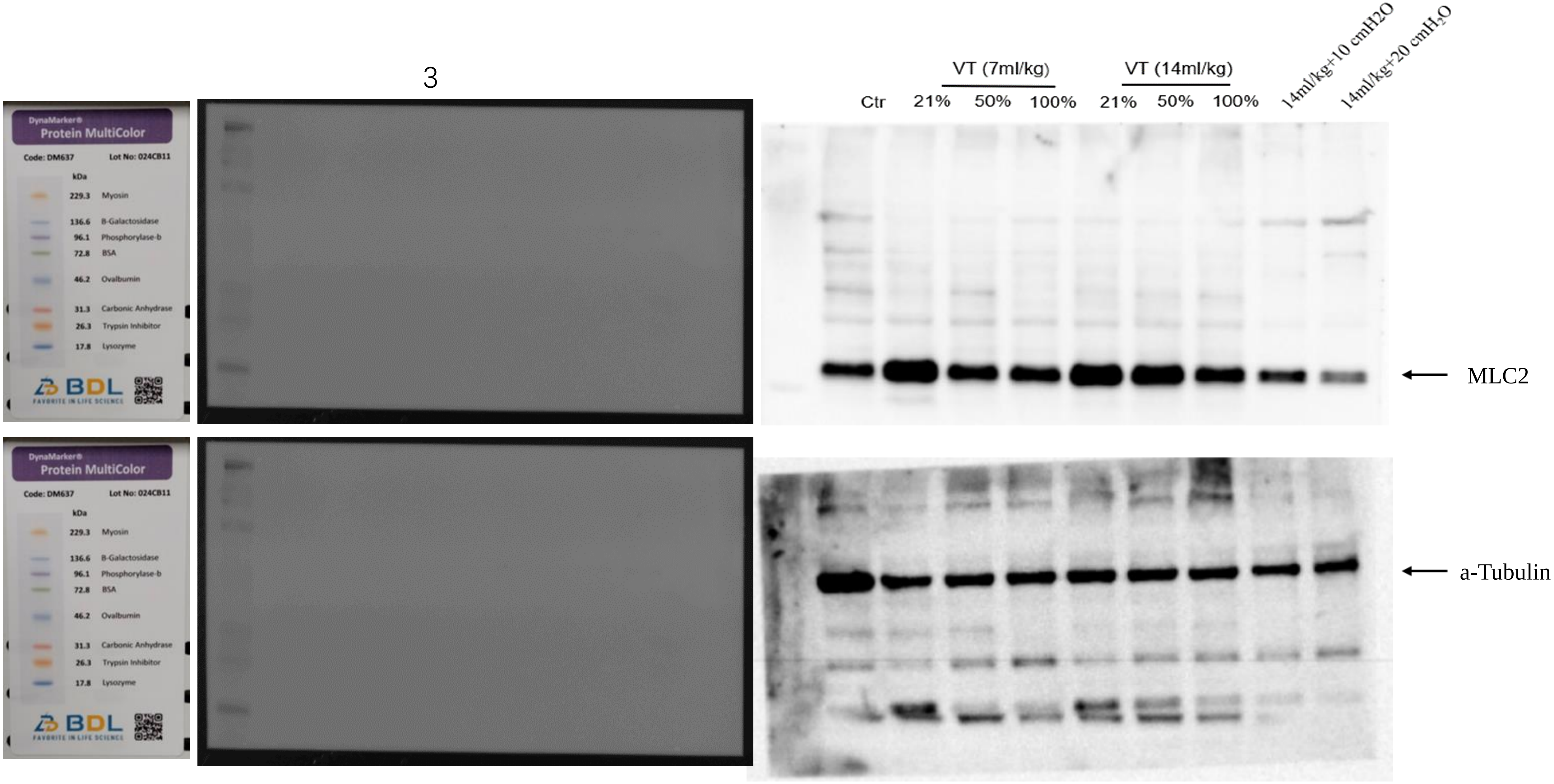
DynaMarker®
Protein MultiColor
Code: DM637
Lot No: 024CB11
kDa
229.3 Myosin
136.6 B-Galactosidase
96.1 Phosphorylase-b
72.8 BSA
46.2 Ovalbumin
31.3 Carbonic Anhydrase
26.3 Trypsin Inhibitor
17.8 Lysozyme
BDL
FAVORITE IN LIFE SCIENCE
VT (7ml/kg)
VT (14ml/kg)
14ml/kg+10 cmH2O
14ml/kg+20 cmH$_2$O
Ctr
21%
50%
100%
21%
50%
100%
MLC2
a-Tubulin

Supplementary figure 4d: p-MLC2

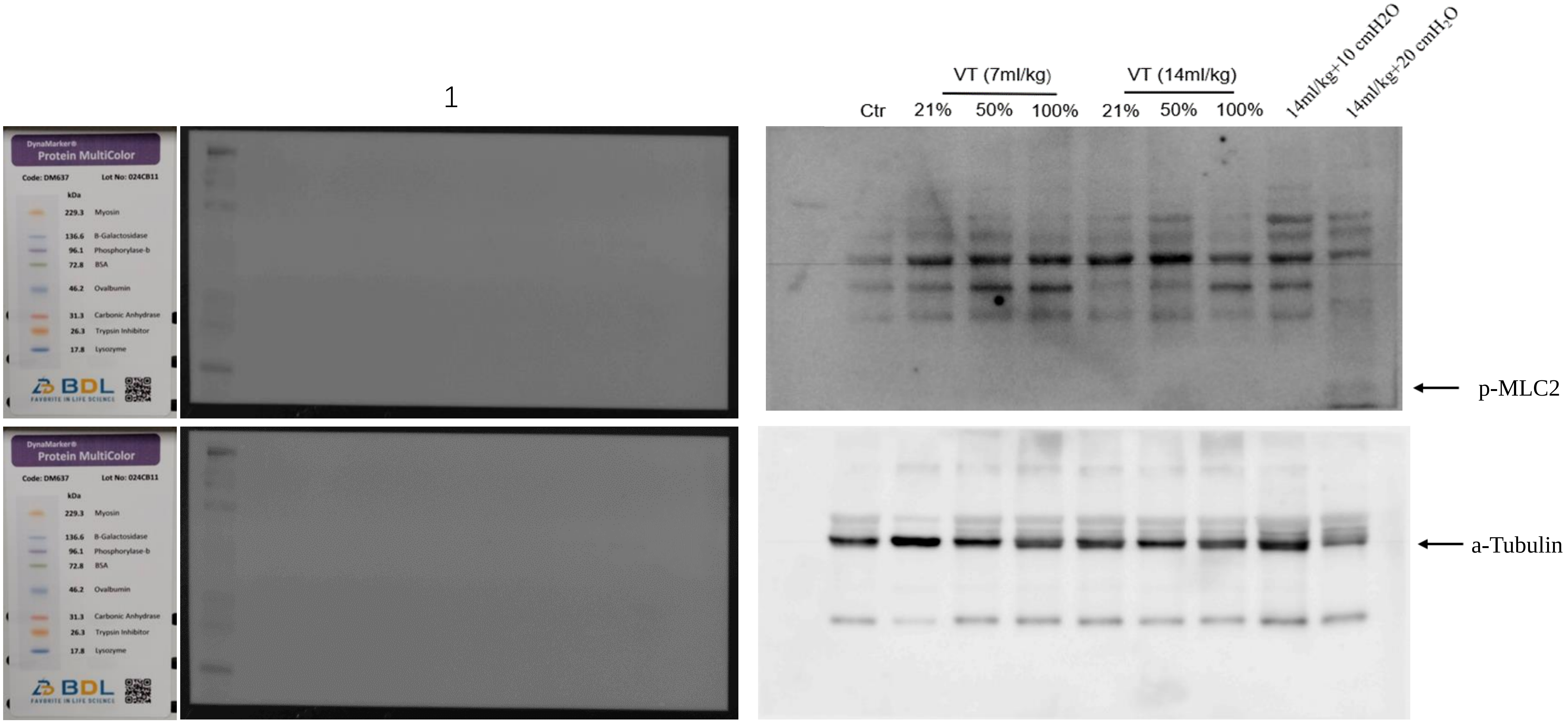
1
DynaMarker®
Protein MultiColor
Code: DM637
Lot No: 024CB11
kDa
229.3 Myosin
136.6 B-Galactosidase
96.1 Phosphorylase-b
72.8 BSA
46.2 Ovalbumin
31.3 Carbonic Anhydrase
26.3 Trypsin Inhibitor
17.8 Lysozyme
BDL
VT (7ml/kg)
VT (14ml/kg)
Ctr
21%
50%
100%
21%
50%
100%
14ml/kg+10 cmH2O
14ml/kg+20 cmH$_2$O
p-MLC2
a-Tubulin

2

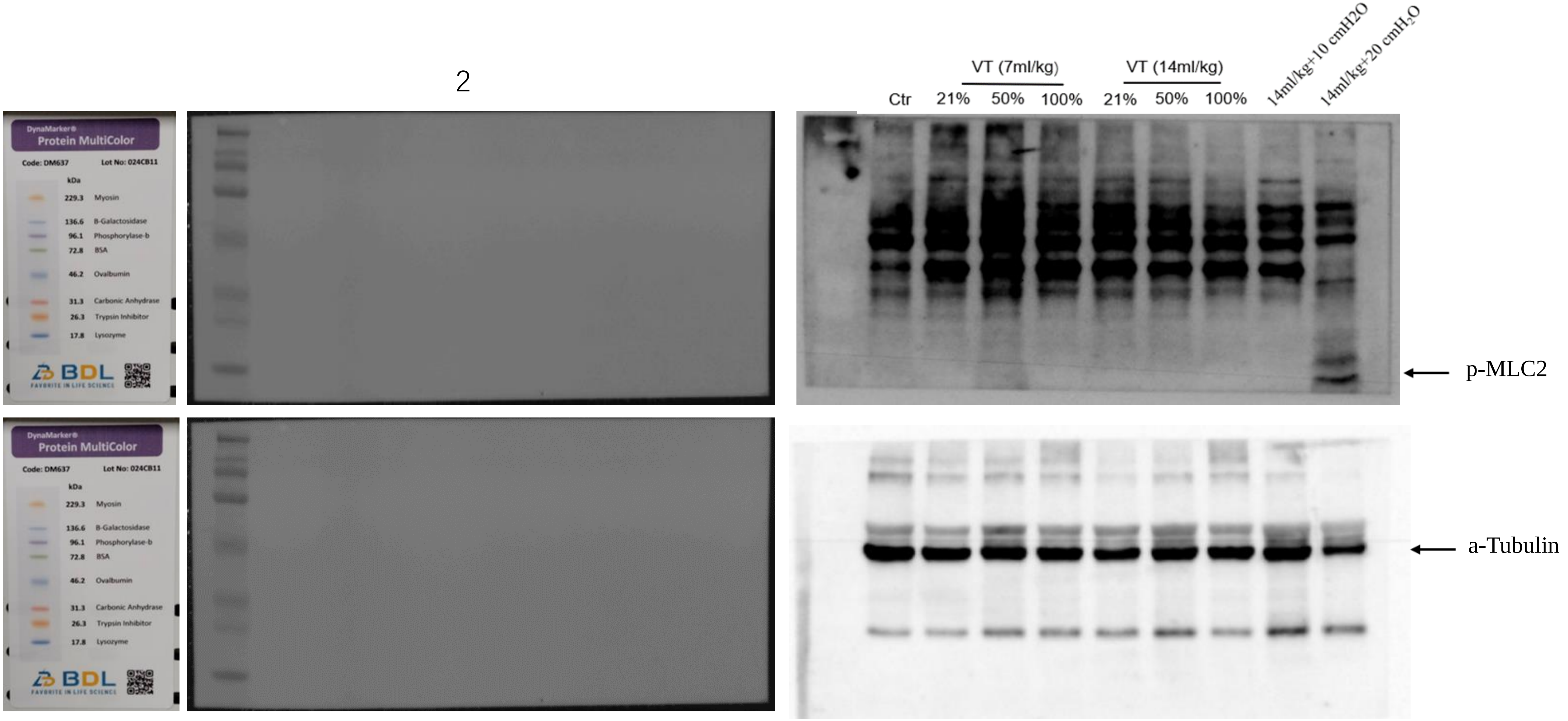
DynaMarker®
Protein MultiColor
Code: DM637
Lot No: 024CB11
kDa
229.3 Myosin
136.6 B-Galactosidase
96.1 Phosphorylase-b
72.8 BSA
46.2 Ovalbumin
31.3 Carbonic Anhydrase
26.3 Trypsin Inhibitor
17.8 Lysozyme
BDL
FAVORITE IN LIFE SCIENCE
VT (7ml/kg)
VT (14ml/kg)
Ctr
21%
50%
100%
21%
50%
100%
14ml/kg+10 cmH2O
14ml/kg+20 cmH$_2$O
p-MLC2
a-Tubulin

Supplementary figure 1d:8-OHdG

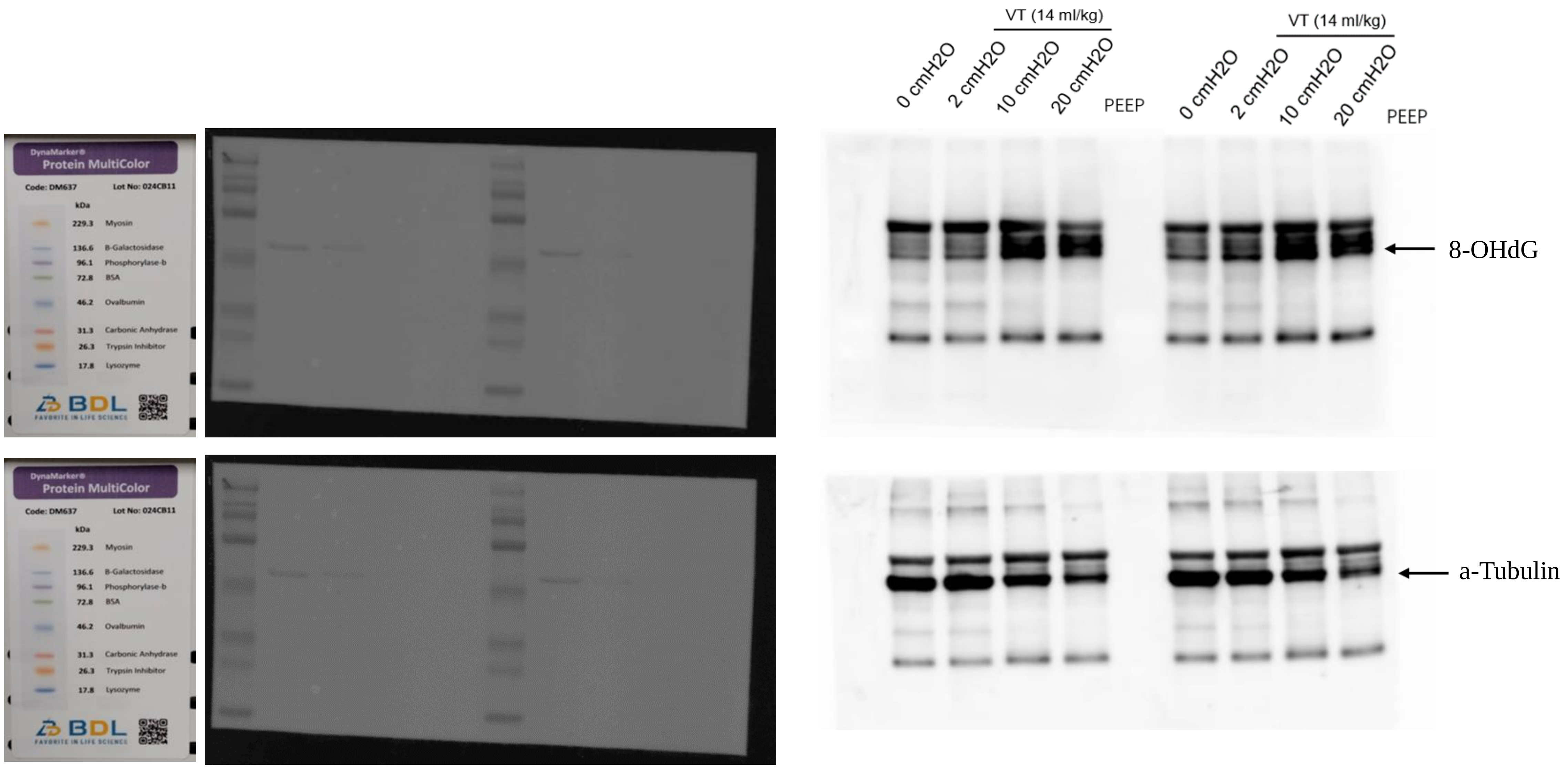
DynaMarker®
Protein MultiColor
Code: DM637
Lot No: 024CB11
kDa
229.3 Myosin
136.6 B-Galactosidase
96.1 Phosphorylase-b
72.8 BSA
46.2 Ovalbumin
31.3 Carbonic Anhydrase
26.3 Trypsin Inhibitor
17.8 Lysozyme
BDL
FAVORITE IN LIFE SCIENCE
VT (14 ml/kg)
0 cmH2O
2 cmH2O
10 cmH2O
20 cmH2O
PEEP
VT (14 ml/kg)
0 cmH2O
2 cmH2O
10 cmH2O
20 cmH2O
PEEP
8-OHdG
a-Tubulin
